# Large Room Temperature Bulk DNP of ^13^C via P1 Centers in Diamond

**DOI:** 10.1021/acs.jpcc.2c06145

**Published:** 2022-10-03

**Authors:** Daphna Shimon, Kelly A. Cantwell, Linta Joseph, Ethan Q. Williams, Zaili Peng, Susumu Takahashi, Chandrasekhar Ramanathan

**Affiliations:** † Institute of Chemistry, The Hebrew University of Jerusalem, Edmond J. Safra, Givat Ram, Jerusalem9190401, Israel; ‡ Department of Physics and Astronomy, Dartmouth College, Hanover, New Hampshire03755, United States; § Department of Chemistry, University of Southern California, Los Angeles, California90089, United States; ∥ Department of Physics and Astronomy, University of Southern California, Los Angeles, California90089, United States

## Abstract

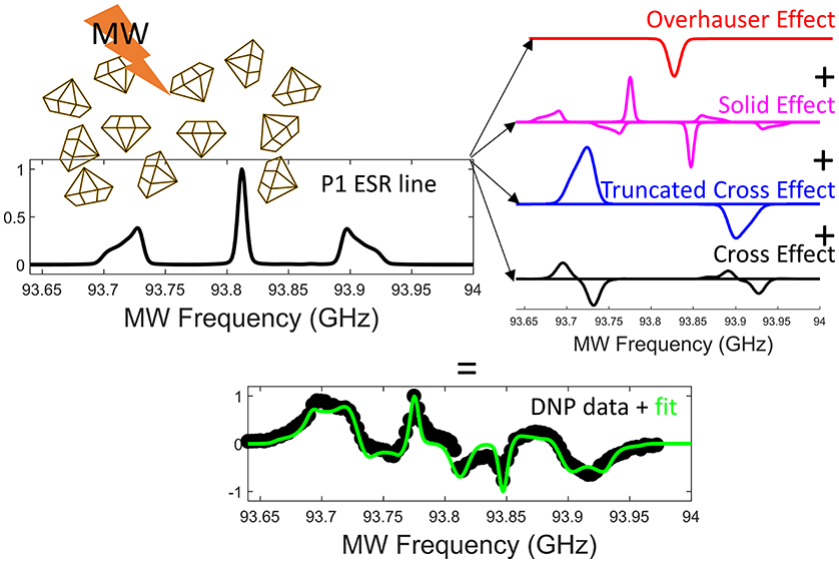

We use microwave-induced dynamic nuclear polarization (DNP) of the substitutional nitrogen defects (P1 centers) in diamond to hyperpolarize bulk ^13^C nuclei in both single crystal and powder samples at room temperature at 3.34 T. The large (>100-fold) enhancements demonstrated correspond to a greater than 10 000-fold improvement in terms of signal averaging of the 1% abundant ^13^C spins. The DNP was performed using low-power solid state sources under static (nonspinning) conditions. The DNP spectrum (DNP enhancement as a function of microwave frequency) of diamond powder shows features that broadly correlate with the EPR spectrum. A well-defined negative Overhauser peak and two solid effect peaks are observed for the central (*m*_*I*_ = 0) manifold of the ^14^N spins. Previous low temperature measurements in diamond had measured a positive Overhauser enhancement in this manifold. Frequency-chirped millimeter-wave excitation of the electron spins is seen to significantly improve the enhancements for the two outer nuclear spin manifolds (*m_I_* = ±1) and to blur some of the sharper features associated with the central manifold. The outer lines are best fit using a combination of the cross effect and the truncated cross effect, which is known to mimic features of an Overhauser effect. Similar features are also observed in experiments on single crystal samples. The observation of all of these mechanisms in a single material system under the same experimental conditions is likely due to the significant heterogeneity of the high pressure, high temperature (HPHT) type Ib diamond samples used. Large room temperature DNP enhancements at fields above a few tesla enable spectroscopic studies with better chemical shift resolution under ambient conditions.

## 1 Introduction

Nuclear magnetic resonance (NMR) spectroscopy shows exquisite chemical sensitivity when reporting on the local magnetic environments of the spins at atomic scales. However, its low detection sensitivity has long required the use of relatively large sample volumes. Microwave-induced dynamic nuclear polarization (DNP), a technique to produce large nuclear spin signal enhancements via polarization transfer from electrons,^1−4^ is driving a technological revolution by enabling NMR and magnetic resonance imaging (MRI) studies of low abundance, low-γ spins and nuclei at surfaces and interfaces for the first time.^5,6^

The electron–nuclear polarization transfer step in most DNP experiments is typically performed at cryogenic temperatures in order to reduce the electron spin–lattice relaxation rates below the strength of the hyperfine interactions. This is true both for dissolution DNP, which is increasingly being used to study room-temperature phenomena in biomedical systems,^7^ and for high-field magic-angle sample spinning (MAS) DNP which has enabled high-sensitivity, high-resolution NMR studies of chemistry in sample-limited solid systems.^5^ Large room temperature DNP enhancements at high field would enable high-resolution NMR spectroscopic studies under ambient conditions.

The nitrogen-vacancy (NV) center in diamond is a promising electron spin system for room temperature DNP applications at low field because of its long coherence and relaxation times.^8−12^ The electron spin of the NV center can be optically polarized to near unity polarization at room temperature. NV centers can also be detected (even at the single spin level) and coherently manipulated using optically detected magnetic resonance (ODMR). In addition to hyperpolarizing the ^13^C spins locally around a single NV center,^13−15^ the hyperpolarization of bulk samples via ensembles of NV centers has been demonstrated, both at low^16−18^ and high magnetic fields.^19−21^ However, at high magnetic fields, the magnitude and sign of the ^13^C hyperpolarization were seen to depend strongly on the orientation of the diamond crystal,^21^ making it difficult to hyperpolarize bulk powders.

Nanodiamonds are chemically stable and their surfaces can be chemically functionalized, making them excellent candidates as local NMR probes^22,23^ (similar to silicon particles^24−27^) as well as fluorescent biomarkers.^9^ A key challenge to the practical use of DNP via defects in diamond is the need to achieve high polarizations outside the diamond surface. While the hyperpolarization of spins on the surface of diamond samples has been demonstrated at low magnetic fields,^28−31^ the transfer of polarization to external spins remains challenging. Relaxation due to surface defects and slow spin diffusion in natural abundance ^13^C samples have so far been the key limiting factors to achieving high polarizations.

In this work we study microwave-induced DNP of diamond at room temperature at 3.34 T under static (nonspinning) conditions. The P1 center is a spin-1/2 substitutional nitrogen impurity in the diamond lattice that also exhibits long coherence and relaxation times,^32−34^ though it is not optically active. There are typically at least an order of magnitude more P1 centers in a diamond crystal than NV centers,^34^ though the best efficiencies for converting P1 to NV centers can reach up to 20–25%.^35,36^ As a consequence, most ^13^C nuclear spins in diamond are much closer to a P1 center than an NV center, making the P1 centers potentially more efficient polarization sources. At high magnetic fields, DNP via P1 centers can produce a significant increase in nuclear spin polarization at cryogenic temperatures.^32,37−41^ Bretschneider et al. have also reported a room temperature ^13^C DNP enhancement of 130 at 9.4 T under 8 kHz MAS conditions with 10 W of microwave power at 263 GHz.^40^ Other high-field room temperature enhancements have also been reported.^42^ Hyperpolarization via P1 centers is also well suited to applications when optical access is not possible.

Here, we show a greater than 100-fold enhancement of the ^13^C spins in both single crystal and powder diamond samples. In contrast to earlier work, these enhancements were achieved using a solid-state source with about 240 mW of power. The DNP spectrum exhibits multiple features indicating that several different DNP mechanisms are operational in these systems. We elucidate the different physical mechanisms and explain their role in both single crystal and powder samples of diamond. Understanding these mechanisms could guide the design of more effective hyperpolarization strategies and the improved design of diamond substrates for polarizing external spins.

The paper is organized as follows: Following a description of the materials and methods in section 2, our main experimental results on the powder sample are described in section 3.1. We present a brief overview of the relevant DNP mechanisms in section 3.2 and discuss the fit of these mechanisms to the observed DNP spectra for both the single crystal and powder samples in section 3.3. Finally, we discuss how sample heterogeneity gives rise to the different mechanisms in section 3.4 and conclude in section 4.

## 2 Materials and Methods

### 2.1 Samples

We used both powder and single crystal diamond samples in these experiments. The bulk diamond powder was donated by Element 6. The type Ib diamond is made by high pressure, high temperature (HPHT) synthesis. The diamond microparticles are 15–25 μm in diameter and are specified to have a nitrogen concentration less than 200 ppm (Element 6 estimated the actual values were about 110–130 ppm). The single crystal macle-cut HPHT diamond samples used were purchased from Element 6, with a specified nitrogen concentration less than 200 ppm.

### 2.2 DNP Experiments

The DNP experiments were performed on a home-built DNP spectrometer, at a field of 3.34 T, corresponding to an electron Larmor frequency of 94 GHz, a ^1^H Larmor frequency of 142 MHz, and a ^13^C Larmor frequency of 35.8 MHz.^43^ All experiments were performed at room temperature. The NMR pulses and detection were controlled with a Bruker Avance AQX spectrometer. More details about the W-band millimeter wave system are provided in section I of the Supporting Information. DNP spectra were recorded by keeping the 1.012 GHz VCO settings constant and stepping the 4 GHz source in order to cover a range of 93.63–93.972 GHz. Data were typically taken over 102 evenly spaced frequencies points.

The following settings were used for all the experiments, unless otherwise noted: The DNP enhanced NMR signal was recorded with a 90-acquire pulse sequence, using a 10 μs π/2 pulse. Eight-step phase cycling was used in all cases during signal averaging. The experiments began with a train of 100 30 ms saturation pulses separated by 20 μs.

The ^13^C relaxation time *T*_1_ (section VI of the Supporting Information) was recorded using saturation-recovery experiments, stepping the recovery delay and then recording the signal, with no MW irradiation applied. The DNP enhancement buildup time *T*_bu_ was recorded in the same manner as *T*_1_ but with MW irradiation.

### 2.3 NMR Data Processing

Data processing was performed in MATLAB using custom scripts. A three-point left-shift was used in all cases to remove the switching transient from opening of the receiver. The data were then baseline corrected and phase corrected, and a 300 Hz exponential line broadening was applied. For DNP enhancement calculations we divide the integrated intensity of the MW-on signal from the MW-off signal (such that no enhancement is equal to 1). The MW-off signal was measured using 128 scans except at the longest buildup times when 64 scans were used.

The reported signal intensities correspond to an average over 21 points around the peak of the phased absorptive signal in the frequency domain. The reported uncertainties in the NMR signal were calculated using the standard deviation (201 point interval) from a signal-free region of the NMR spectrum.

The *T*_1_ and *T*_bu_ curves (sections VI and VIII in the Supporting Information) were fit using a biexponential function, using the method of a nonlinear least-squares. The fitting function returns a 95% confidence interval which was converted to a variation of ±2σ assuming a normal distribution.

The ^13^C NMR spectrum was referenced to adamantane, at 37 ± 1 ppm using glycerol-*d*_5_ as a secondary reference, with two peaks at 64 ± 1 and 72 ± 1 ppm.

### 2.4 EPR Experiments

EPR experiments were conducted at both high and low magnetic field.

#### 2.4.1 High-Field CW EPR

Room-temperature continuous-wave (cw) electron paramagnetic resonance (EPR) lines were measured at 230 GHz at the University of Southern California. The experiments used a field modulation frequency of 20 kHz and a modulation strength of ∼0.02 mT.

#### 2.4.2 Low Field Pulsed EPR

Our lab-built pulse-EPR spectrometer is designed to operate at 2.5 GHz and can output up to 1 W of power. Additional details can be found in section X of the Supporting Information. Hahn-echo and inversion recovery experiments were conducted on the powder sample and on the single crystal diamond with **B**_0_ parallel to [111]. The pulsed data were acquired on the *m*_*I*_ = 0 transition of the EPR line.

#### 2.4.3 Pulsed EPR Data Processing

For the Hahn-echo experiment, the sequence is π/2−τ–π–τ–echo, and the interpulse delay τ is swept. The magnitudes of the echoes are plotted as a function of the time at which the echo comes into focus. For both the powder and the single crystal diamond, the Hahn-echo magnitudes *S*_HE_(*t*_E_) are fit to a biexponential decay equation,
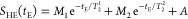
1where *t*_E_ = 2τ + *t*_π/2_/2 + *t*_π_ is the cumulative time that spins are undergoing *T*_2_ relaxation, *M*_1_ and *T*_2_^1^ are the amplitude and transverse relaxation time characteristic of electrons in pool 1, *M*_2_ and *T*_2_^2^ are the likewise variables for pool 2, and *A* is an offset that is necessarily nonzero since the echo magnitudes are used in fitting. Similarly, the inversion recovery magnitudes *S*_IR_(*t*_E_) can also be fit to a biexponential decay equation,
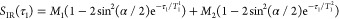
2where *M*_1_ and *T*_1_^1^ are the amplitude and longitudinal relaxation time characteristic of electrons in pool 1, *M*_2_ and *T*_1_^2^ are the likewise variables for pool 2, and α is an angle whose deviation from π indicates imperfect inversion of the electron spin population.

## 3 Results and Discussion

### 3.1 Room Temperature DNP via P1 Centers

**Figure 1:**
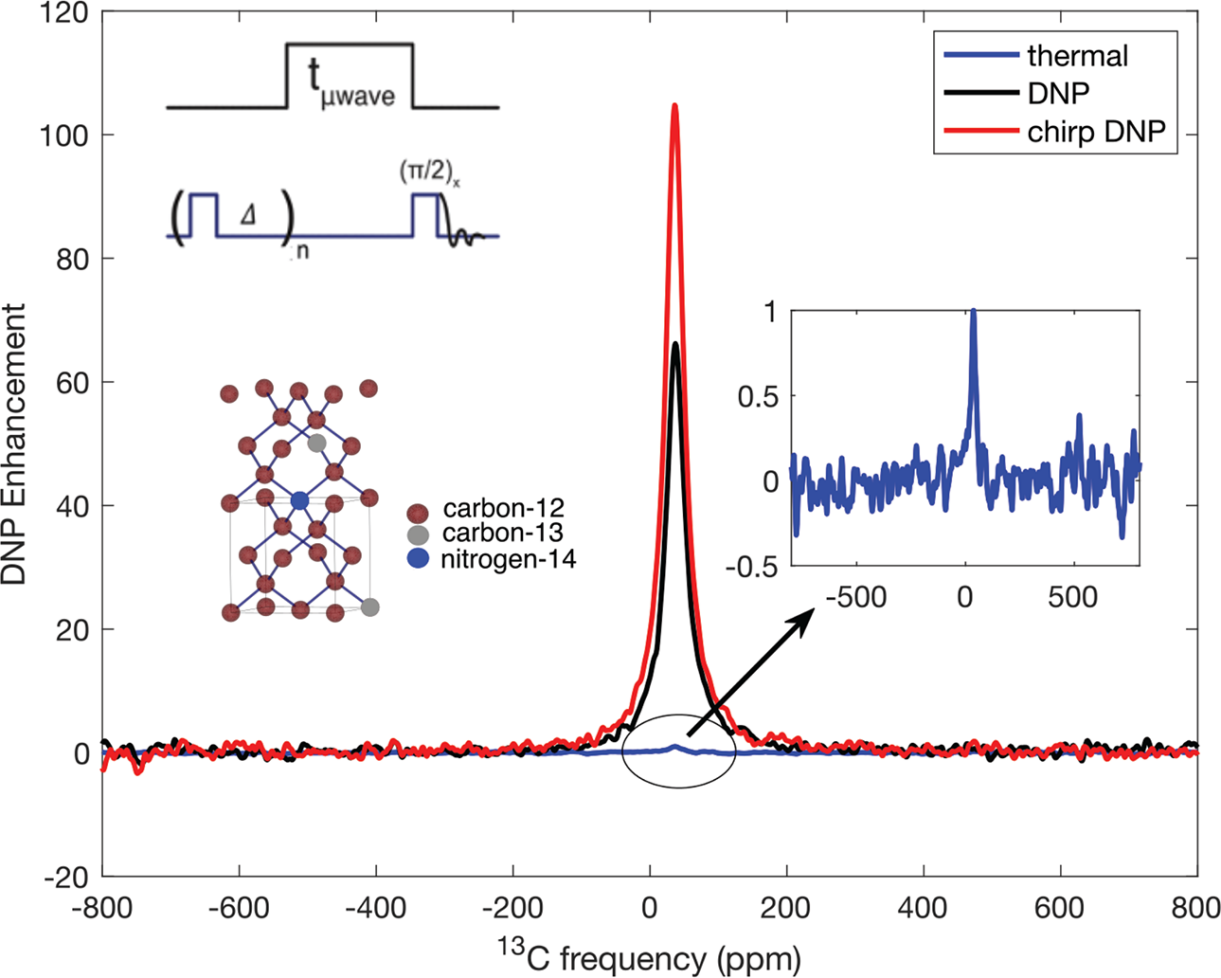
^13^C NMR spectrum of the diamond powder, with MW irradiation (black line) at 93.696 GHz and without MW irradiation (blue line), resulting in DNP enhancement of 75. With the chirped excitation the enhancement is seen to rise to 114 (red line). The signal was recorded after a recycle delay of 3000 s for the thermal signal and after 3000 s of MW irradiation for the DNP and chirp DNP signals. Triangular ramp-up modulation was used with a 5 kHz modulation frequency and a 117 MHz modulation amplitude. The pulse sequence used to record the NMR spectra is shown in the inset, with Δ being the delay in the saturation pulse train and *t*_μwave_ the DNP buildup time. The model of the diamond lattice shown in the figure was created using VESTA.^44^

Figure 1 shows the thermal equilibrium ^13^C signal from a bulk diamond powder sample after 128 averages with a recycle delay of 3000 s. The signal was acquired using the pulse sequence shown in the inset of Figure 1.

The figure also shows the single shot DNP signal with the same 3000 s buildup time under constant frequency irradiation and chirped millimeter-wave (MW) irradiation centered around the frequency of 93.696 GHz. At this MW excitation frequency the constant frequency DNP excitation resulted in a DNP enhancement of 75 ± 8 and the chirped excitation results in a DNP enhancement of 114 ± 11. The chirped excitation used a triangular ramp-up function with a 5 kHz modulation frequency and a 117 MHz modulation amplitude. The maximum output power of our MW source is about 240 mW.

All NMR spectra show a single peak at 37 ± 1 ppm, which matches the literature value for ^13^C nuclei in diamonds^32^ (see Figure 1). The NMR peak has a width of 1.12 kHz showing significant inhomogeneous line-broadening. It is possible to detect several hundred echoes in a pulsed spin-lock experiment as described in section II of the Supporting Information. Stroboscopic detection of multiple echoes would significantly improve the signal-to-noise ration (SNR).

**Figure 2:**
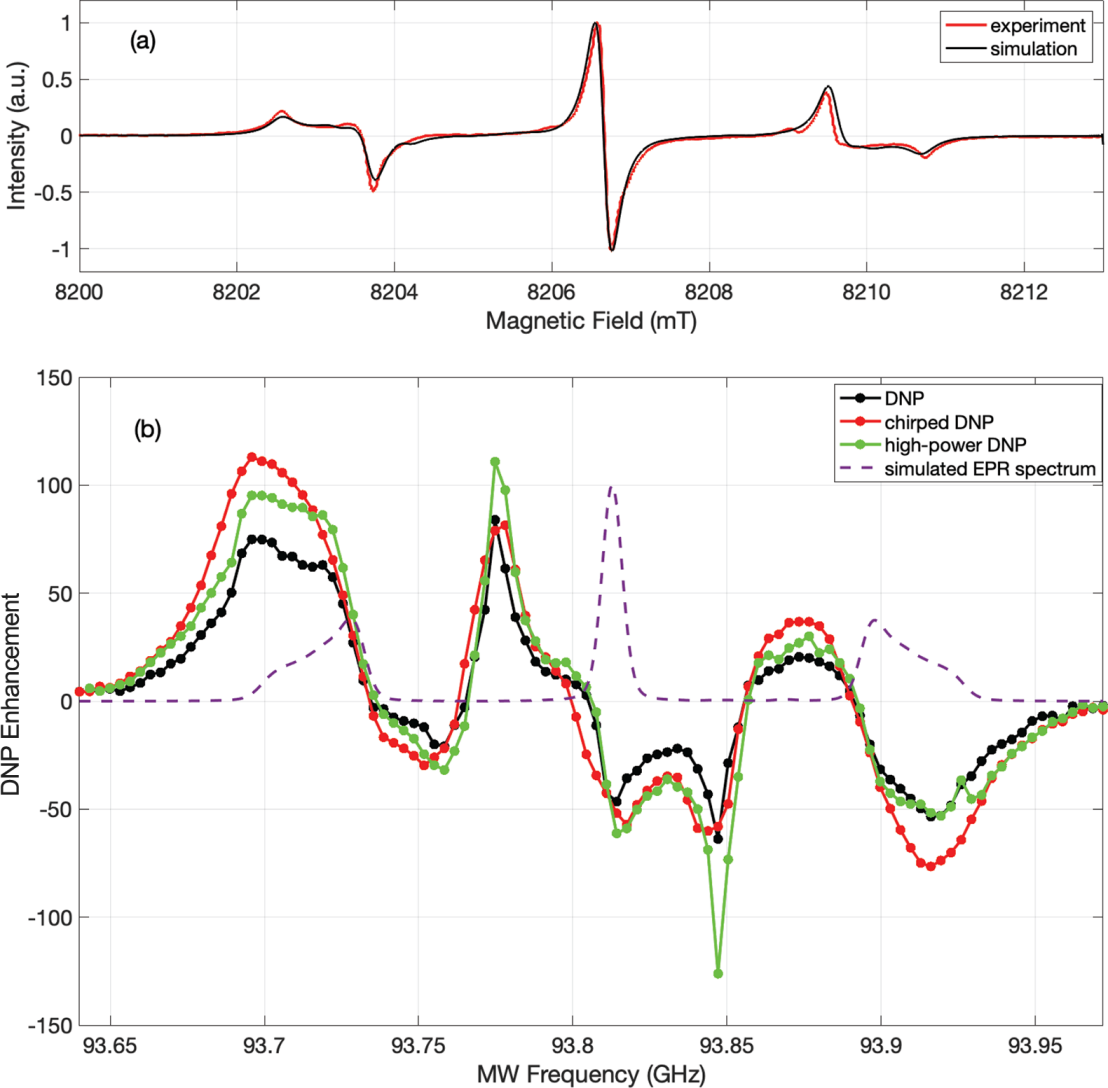
(a) High-field EPR spectrum of the diamond powder and the best fit according to EasySpin.^45^ The simulation of the experimental EPR line used a 0.19 mT Lorentzian line broadening. (b) ^13^C DNP spectrum of the diamond powder: enhancement measured as a function of constant frequency MW (black symbols) and chirped frequency MW irradiation (red symbols) with 240 mW power, and constant frequency MW at 500 mW power (green symbols). The figure also shows simulated EPR spectrum under the experimental conditions (dashed magenta line). The simulation of the expected EPR line at 94 GHz used a 6 MHz line broadening. Error bars are not shown (the ∼10% errors in enhancement are dominated by the SNR of the thermal signal).

Figure 2a shows the experimentally measured continuous-wave (CW) EPR spectrum of the sample measured at room temperature using the 230 GHz EPR system at the University of Southern California.^46,47^ The EPR spectrum features three lines, consisting of a single electron split by the hyperfine coupling to the spin-1 ^14^N nucleus of the P1 center. The anisotropic hyperfine interaction results in a powder broadening of the *m*_*I*_ = ±1 manifolds. The figure also shows an EasySpin^45^ quantum mechanical simulation of the spectrum for this sample overlaid on the experimental spectrum. Each P1 center was modeled as an e-^14^N system, with an isotropic *g*-factor, *g* = 2.0024, hyperfine coupling strengths with the ^14^N nucleus with principal axis components *A*_*x*_^N^ = *A*_*y*_^N^ = 82 MHz, *A*_*z*_^N^ = 114 MHz ^48^ (see section IV of the Supporting Information). The ^14^N nuclear quadrupolar interaction was neglected.

Figure 2b shows the DNP spectrum (DNP enhancement as a function of millimeter wave frequency) for ^13^C obtained with a buildup time of 3000 s under three different experimental conditions. These include (i) constant frequency MW excitation with ∼240 mW power, (ii) frequency-chirped MW excitation with ∼240 mW power using the same triangular ramp-up function described above, and (iii) constant frequency MW excitation with ∼500 mW power. The higher power sweep was obtained by using the attenuated output of the 240 mW source to injection-lock an IMPATT diode source. The figure also shows the simulated EPR spectrum at 3.34 T using the same sample parameters used to fit the high-field spectra. The estimated uncertainty in the enhancement is about 10%, which is dominated by the standard deviation of the thermal signal.

### 3.2 DNP Mechanisms

**Figure 3:**
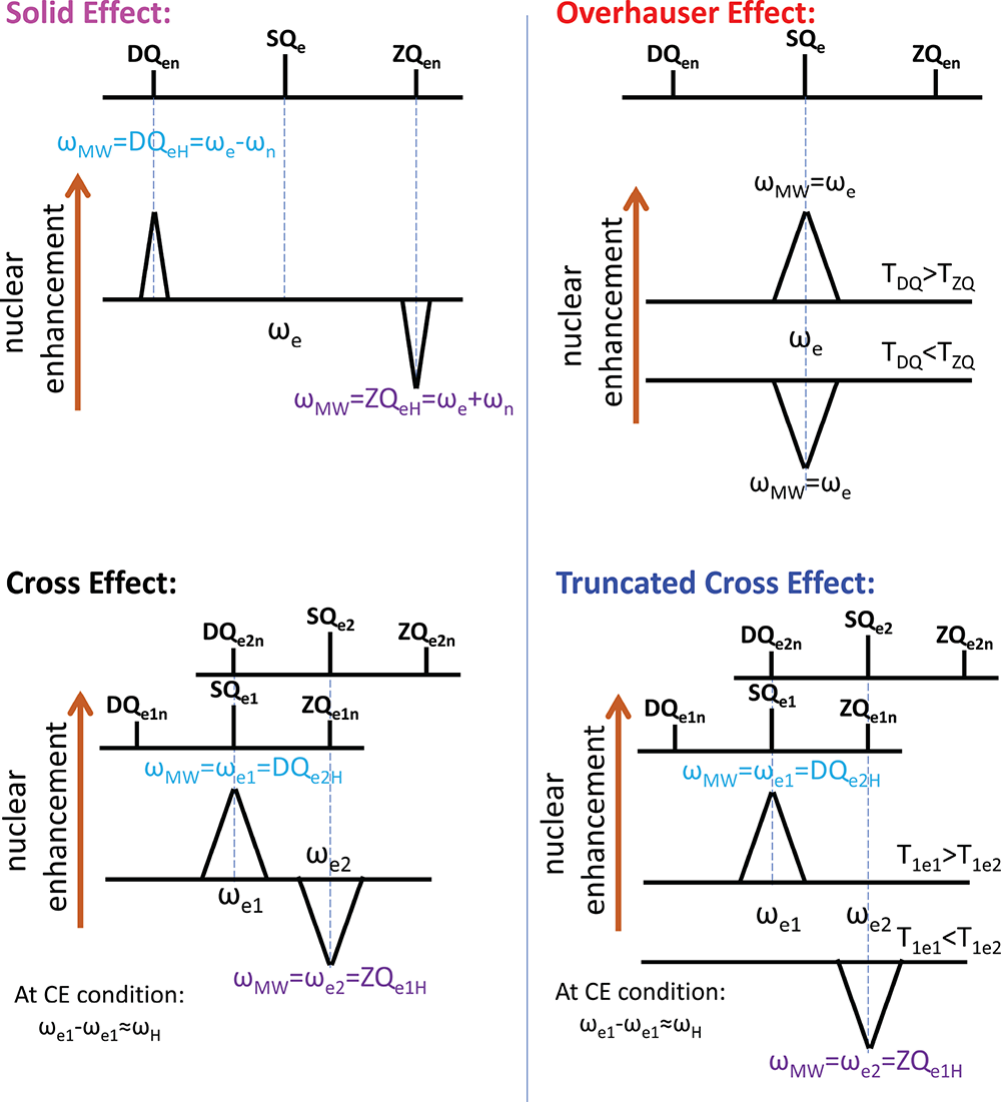
Schematic DNP enhancement spectra showing the positions of the SE-DNP (e–n system), OE-DNP (e–n system), CE-DNP (e–e–n system), and truncated CE-DNP (e–e–n system) mechanisms with respect to the EPR transitions in each system. The DQ and ZQ transition labels represent a nucleus with a positive gyromagnetic ratio, and an electron with a negative gyromagnetic ratio.

While the DNP spectrum in Figure 2b is seen to broadly correlate with the EPR spectrum, it is not immediately possible to identify the underlying DNP mechanism(s). Here we outline the different DNP mechanisms that could play a role. Most solid systems studied by DNP typically exhibit hyperpolarization via one of five mechanisms.^1,2,49,50^Figure 3 schematically illustrates the first four mechanisms. In broad EPR lines, positive and negative enhancements from the same mechanism and different mechanisms often overlap.^3,51,52^ Additional smaller DNP features have also been observed due to the higher-order multispin processes.^53^

#### 3.2.1 Overhauser Effect (OE)

The OE is typically observed in metals and liquids, systems in which the hyperfine interactions are strongly modulated in time. It has been shown, however, that the OE can also be observed in dielectric systems with strong localized exchange interactions^54^ and in mixed-valence organic radicals such as BDPA.^55−58^ Fluctuations of the hyperfine interaction result in electron–nuclear (e–n) cross-relaxation at the double quantum (DQ, ω_e_ – ω_n_) or zero quantum (ZQ, ω_e_ + ω_n_) e–n transitions, with characteristic decay times *T*_1DQ_ and *T*_1ZQ_, respectively.^55−57^ When irradiating directly on the electron single quantum (SQ) transitions, an imbalance between *T*_1DQ_ and *T*_1ZQ_ results in nuclear enhancement, with positive enhancement when *T*_1DQ_ > *T*_1ZQ_ and negative enhancement when *T*_1DQ_ < *T*_1ZQ_.

#### 3.2.2 Solid Effect (SE)

The SE is typically observed in isolated electron–nuclear spin systems in insulators where anisotropic hyperfine interactions admix the nuclear spin states. MW irradiation of the nominally “forbidden” DQ (positive enhancement) and ZQ (negative enhancement) transitions leads to the enhancement of the nuclear spin polarization.^1,49,50^ If the ESR line is inhomogeneously broadened, frequency or field modulation can produce an integrated solid effect where the enhancements of the DQ and ZQ become additive under the appropriate modulation conditions.^59^

#### 3.2.3 Cross Effect (CE)

At higher electron spin concentrations, the three-spin (two electrons + one nucleus) CE process results from microwave irradiation of an inhomogeneously broadened EPR line.^49,50^ The CE-DNP mechanism results in nuclear hyperpolarization when two electrons fulfill the so-called CE condition (ω_e1_ – ω_e2_ = ω_n_ for ω_e1_ > ω_e2_) and have unequal polarizations (usually due to irradiation of one of the electrons).^2,60^

#### 3.2.4 Truncated Cross Effect (tCE)

Recently, Equbal et al. observed that it is possible for the CE to masquerade as an OE when the CE condition is satisfied by two pools of electrons, one with a very fast *T*_1e_ relaxation and the other with much slower *T*_1e_ relaxation.^61^ When this happens, irradiating on the electrons that exhibit slow relaxation results in nuclear enhancement, due to the saturation of these electrons, and the formation of a polarization difference between the two pools of electrons. However, irradiating on the electrons that exhibit fast relaxation does not result in nuclear enhancement because the electrons in the fast relaxing pool cannot be saturated. As a result, only positive or negative enhancement will be observed (depending on the MW frequencies of the fast and slow electrons), and the CE will appear truncated. For this truncated CE, the DNP enhancement is directly observed at the EPR frequency of the electron pool with the slow relaxation.

#### 3.2.5 Thermal Mixing (TM)

Finally, TM is a statistical thermodynamics description for many coupled electron spins and requires MW irradiation directly on a homogeneously broadened EPR line.^49,50^ Note that TM is not expected to be significant at the concentration of P1 centers present in this sample.

### 3.3 Analysis of the DNP Spectra

We characterized the DNP mechanisms operational in both single crystal and powder samples.

#### 3.3.1 Single Crystal

In order to better understand the underlying mechanisms, we first attempted to fit the DNP spectrum obtained from a single crystal HPHT type Ib sample. Fitting the single crystal data should be simpler as we do not observe the effects of powder averaging of the anisotropic nitrogen hyperfine interactions. We were unable to measure a thermal NMR signal in the single crystal sample even with extensive averaging. We estimate a lower bound to the maximum enhancement of about 180 determined by the signal-to-noise of the DNP signal (see section III of the Supporting Information).

**Figure 4:**
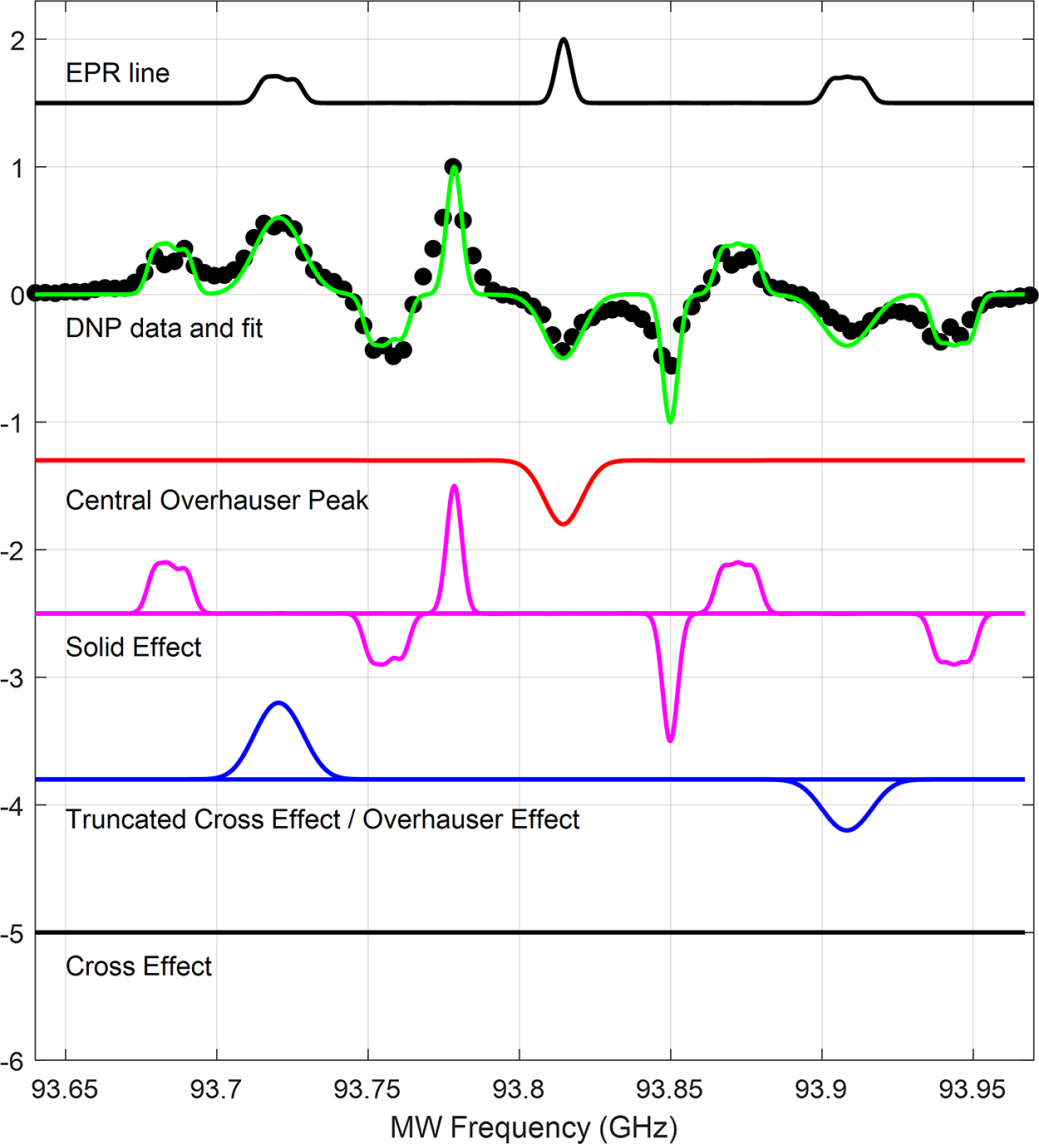
Fit of experimental constant-frequency DNP spectrum (black circles) for a single-crystal diamond sample. The fit uses a sum (green line) of the OE (red line), SE (magenta line), the truncated CE (blue line), and the CE (black line). The EPR line plotted includes line broadening of 10 MHz of the outer EPR lines. The single crystal EPR line was simulated with EasySpin^45^ using the Euler angles α = 54.0013°, β = 134.4274°, γ = 18.0023°.

Figure 4 shows the DNP spectrum obtained from the single crystal sample. The spectrum shows well-resolved peaks in the *m*_*I*_ = ± 1 manifold of the nitrogen spins which allows us to easily simulate the expected EPR spectrum at this crystal orientation. The additional electron of the P1 center lies along one of the four equivalent C–N bonds due to the Jahn–Teller distortion. The EPR spectrum of a single diamond crystal can show between 3 and 7 distinct peaks, depending on the orientation of the crystal with respect to the external field.

In Figure 4, we have fit the DNP spectrum using a very crude method of convolving the EPR line with delta functions to form the basic shapes for the SE, CE, and tCE/OE DNP mechanisms and adjusting their amplitudes to achieve the best agreement with the experimental spectrum.^62^ The shapes were constructed for each EPR line separately, and then the relative amplitudes were adjusted. For a detailed description see section V of the Supporting Information.

In the central nuclear spin manifold (*m*_*I*_ = 0), the sharp features correspond to an OE peak at 93.81 GHz and SE peaks (at the ^13^C nuclear sideband frequencies) at 93.78 and 93.85 GHz.^49,50^ The origin of the temporal modulation that drives OE enhancement is still not clear. One potential source is the dynamic Jahn–Teller distortion. Early ENDOR studies suggested a reorientation rate less than 3.5 GHz at room temperature.^63^ Ammerlaan and Bergmeister studied the reorientation rate of the P1 defect due to thermal excitation and tunneling in the temperature regime between 78 and 200 K,^64^ while Loubser and van Ryneveld studied the rate between 600 and 1230 K.^65^ Interpolating between these sets of experimental results suggests a characteristic reorientation rate on the order of 1–10 Hz at 300 K. This is likely to be too low to drive the observed Overhauser effect as it is necessary to have temporal modulations at the ^13^C Larmor frequency to induce an OE. Another possibility is that the OE is induced by exchange or dipolar-coupled clusters of P1 centers.^54,66^

The outer lines are best fit using the OE or truncated CE. If the mechanism is in fact the OE, the experimental spectrum necessitates that the OE on the low frequency side be positive while the OE on the high frequency side be negative. To our knowledge, this type of effect has never been reported previously, and the origin of the difference in sign is unclear. Therefore, we also suggest that we may be in fact observing two truncated CE peaks.

The truncated CE is possible in this system if we have an additional pool of very fast relaxing P1 centers that appear in the frequency range between the outer manifolds (93.76–93.89 GHz). If this were the case, then irradiating on the low frequency EPR line should result in positive enhancement (because we would be irradiating on the lower frequency EPR line of the CE pair), and irradiating on the high frequency EPR line should result in negative enhancement (because we would be irradiating on the higher frequency EPR line of the CE pair), exactly as observed. These fast relaxing spins would be difficult to observe in a standard CW-EPR experiment.

**Figure 5:**
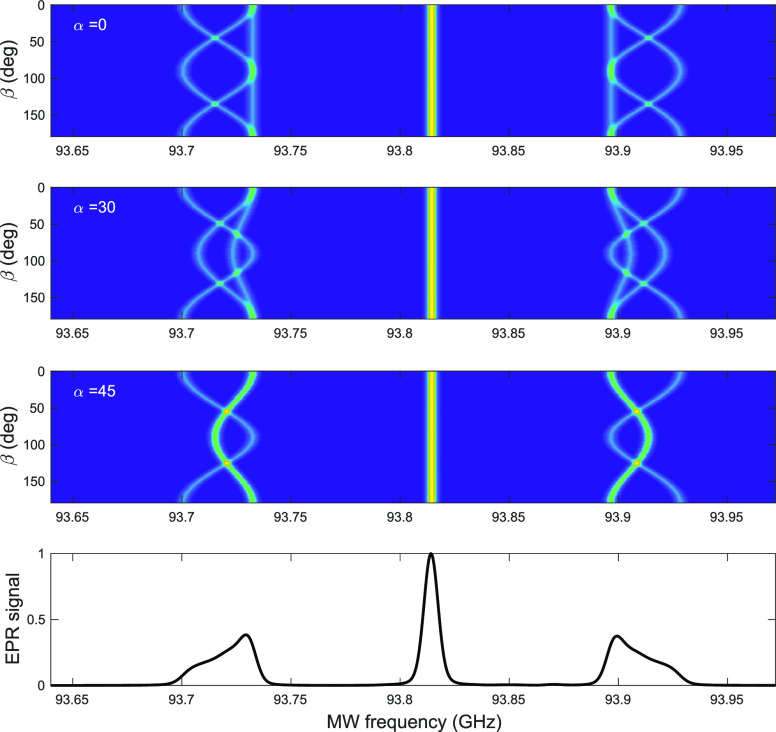
EasySpin simulations^45^ of the EPR spectrum of P1 centers in diamond as a function of crystal orientation. Depending on the orientation, there are up to four distinct orientations of the P1 center with respect to the magnetic field for a single crystal orientation. The Euler angles (α, β, γ) indicate the orientation of the lattice with the external magnetic field. We have set γ = π/4, and the top three subplots indicate rotation patterns as a function of β with α = 0, π/6, and π/4. The bottom curve shows the simulated powder spectrum.

Experimental results from a second crystal orientation exhibiting the same DNP mechanisms are shown in section III of the Supporting Information. While no direct CE is observed at these crystal orientations, this will not necessarily be true for all orientations. In order for a diamond crystal to satisfy the conditions for CE enhancement at a given orientation, there should be two EPR lines separated by the nuclear Larmor frequency. In order to understand the DNP spectrum from the powder, we studied how the EPR spectrum from the diamond sample changes with orientation in the external field. Figure 5 shows EasySpin simulations of how the single crystal spectra change as the crystal is rotated about different axes, as well as the powder averaged spectrum.^45^ There are a number of orientations at which the separation between two of the lines in the *m*_*I*_ = ± 1 manifold is on the order of the ^13^C Larmor frequency. Thus, two adjacent P1 centers (with different relative orientations) can undergo a dipolar-driven mutual spin-flip that would lead to ^13^C DNP. (Other CE conditions may be fulfilled at other fields, such as those described by Bretschneider et al.^40^) Note that the addition of electron–electron dipolar interactions will also cause a broadening of the EPR lines, making the CE condition easier to fulfill.

Section IX of the Supporting Information provides a detailed description of an e–N–C spin system, and full quantum mechanical simulations of the DNP spectra for both ^13^C and ^14^N, including relaxation. These simulations reveal that, in general, the ^13^C-SE-DNP enhancement occurs within a ^14^N manifold (i.e., the nitrogen spin state does not affect the carbon DNP). For the simulations we choose to concentrate on ^13^C-SE-DNP due to its relative simplicity to understand.

#### 3.3.2 Powder

**Figure 6:**
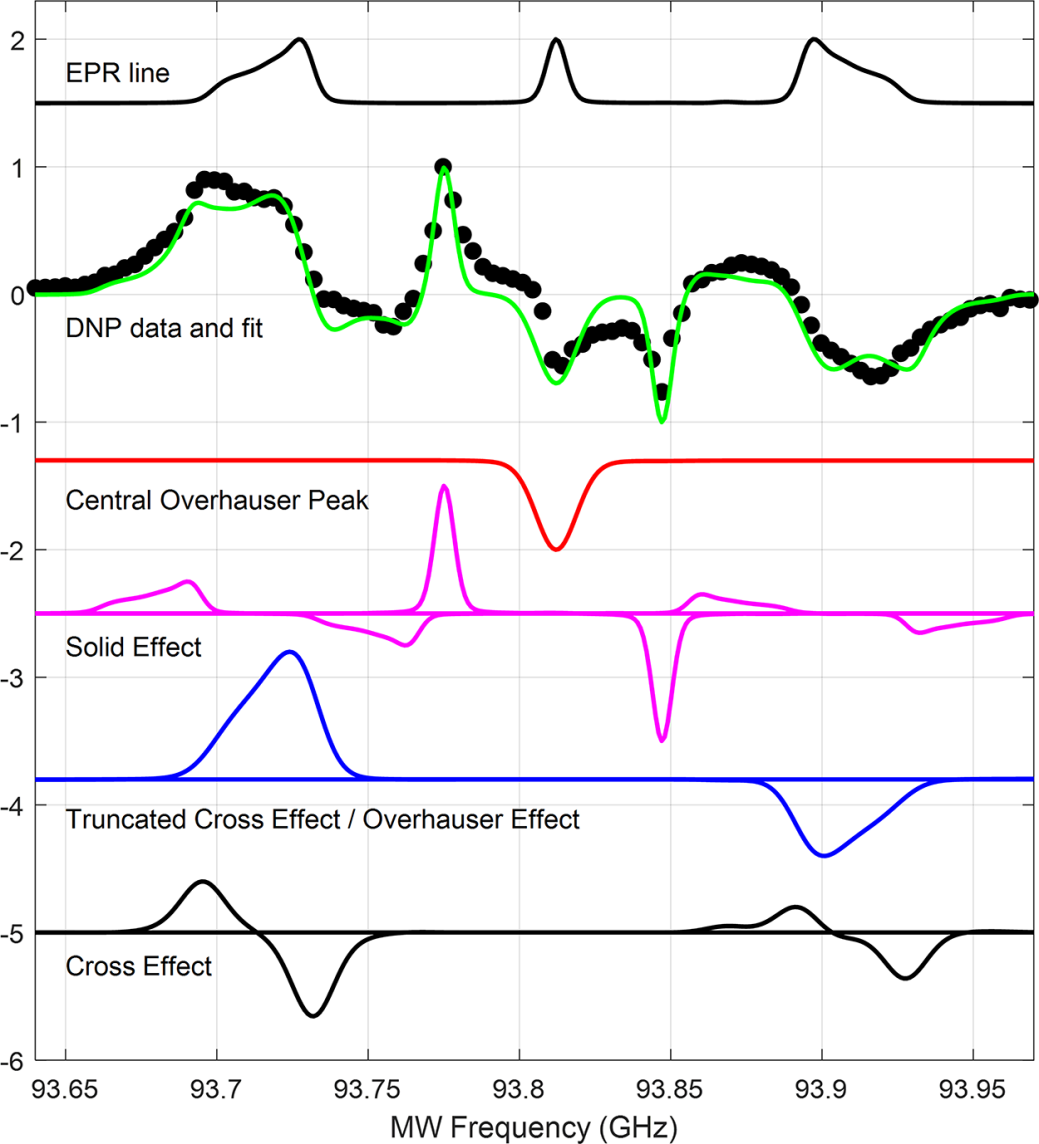
Fit of experimental constant-frequency DNP spectrum (black circles) of the diamond powder sample using a sum (green line) of the OE (red line), SE (magenta line), the truncated CE (blue line), and the CE (black line). The EPR line plotted above includes line broadening of 10 MHz of the outer EPR lines.

Figure 6 shows a fit of the DNP spectrum of the powder sample. The components of the SE, OE, tCE, and CE with their respective intensities are also plotted. As in the single crystal case, the center of the DNP spectrum can be fit using a combination of the SE and the OE giving negative enhancement. Here the outer lines are best fit using a combination of the CE and the OE or truncated CE.

Figure 2b showed that chirped DNP significantly improved the enhancements for the two outer nuclear spin manifolds (*m*_*I*_ = ± 1) and blurred some of the sharper features associated with the central manifold (*m*_*I*_ = 0).^67−69^ The enhancement observed under modulation could be either due to the cross effect^67,68^ or due to the introduction of a new mechanism, the integrated solid effect.^59^ Given the low microwave power and the rapid 5 kHz modulation rate, significantly faster than the electron *T*_1_, it is unlikely that the experiment satisfies the conditions necessary for achieving the integrated solid effect via frequency modulation.^70,71^ Additionally the effect is not observed in the central (*m*_*I*_ = 0) manifold. Thus, the additional enhancement from modulation is believed to be due to the CE.

We have also characterized the power-dependence and the buildup times of the DNP spectrum (see sections VII and VIII of the Supporting Information). Both sets of data suggest that the observed DNP enhancements are limited by the available microwave power.

The plurality of DNP mechanisms observed is more complex than in previous high-field DNP experiments with P1 centers. In a diamond micropowder sample with a P1 concentration less than 100 ppm, Bretschneider et al. observed three SE-DNP lines, one from each EPR line, and a single positive OE line from the central EPR line under static DNP conditions at W-band (94 GHz) and low temperature (1.5–100 K).^40^ Similarly, Kwiatkowski et al. observed static DNP from P1 centers at 3.5 K at both 94 and 196 GHz in nanodiamonds via a combination of the SE and a small positive OE.^41^ The positive OE enhancements measured at low temperature are *opposite* to the negative OE we observe at room temperature. The origin of this difference is still unknown.

### 3.4 Sample Heterogeneity

To our knowledge this is the first case in which the OE, SE, CE, and truncated CE have been observed in a single material system under the same experimental conditions. This is likely due to the heterogeneity in the distribution of nitrogen in these type Ib HPHT diamond samples.

Li et al. recently used double electron–electron resonance (DEER) experiments to show that single crystal HPHT diamond samples (similar to those studied here) show significant spatial variations in their P1 concentrations.^72^ One of the samples they studied was measured to have local P1 concentrations ranging from 13 to 322 ppm in different regions.

**Figure 7:**
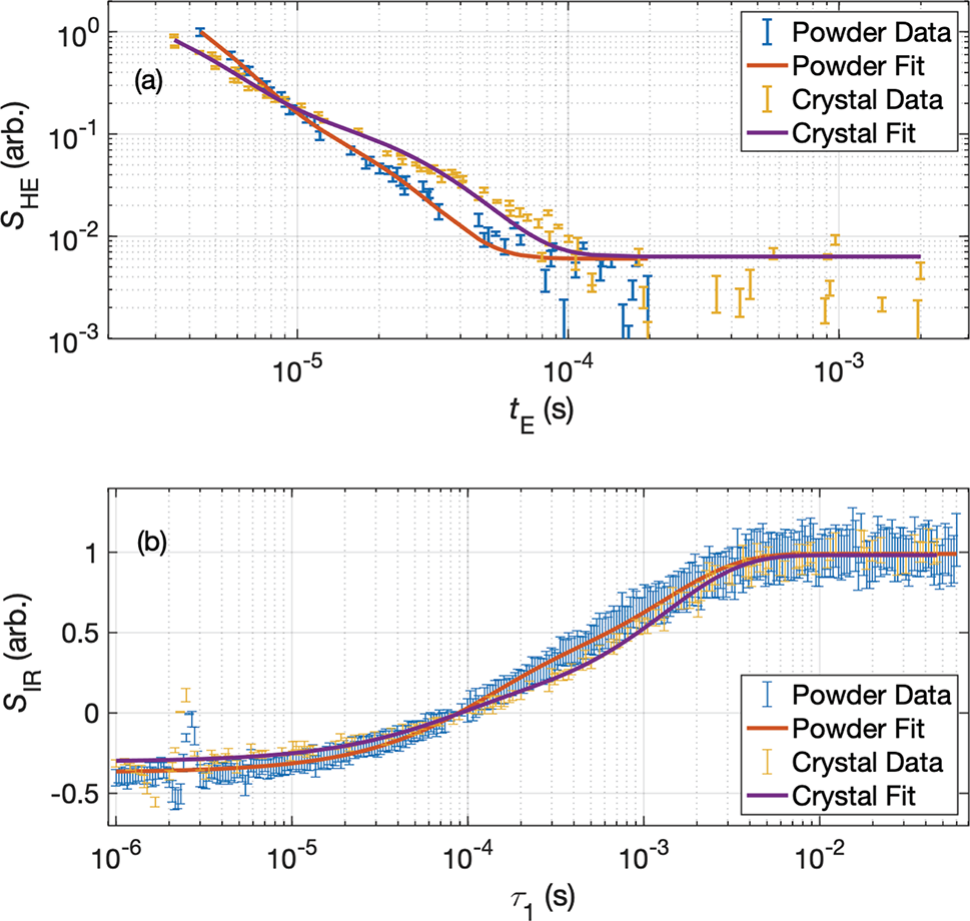
Results from low-field pulse-EPR experiments. The fit parameters are listed in Tables 1 and 2. (a) Hahn-echo decays showing biexponential behavior, which suggests there are at least two types of local environment with different P1 concentrations. (b) Inversion recovery experiment indicating fast and slow electronic T_1_ relaxations. The field dependence is expected to be weak at room temperature as it is likely to be dominated by two-phonon Raman transitions.

We measured the *T*_1_ and *T*_2_ of the P1 center in both our powder sample and a similar single crystal sample to that used for DNP. The 2.5 GHz pulsed EPR setup is described in section X of the Supporting Information. Figure 7 shows that for both the Hahn-echo and the inversion recovery experiments, biexponential relaxation best fits the data (see Tables 1 and 2). The Hahn-echo data for the powder are fit with time constants of about 2 and 10 μs corresponding to ∼80 ppm and ∼16 ppm using the relation 1/*T*_2_ (μs^–1^) = 1/160 (μs^–1^ppm^–1^) × [P_1_] (ppm).^73^ Note that it is difficult to measure *T*_2_ values below 1 μs on our system due to instrument limitations, so there could potentially be pools with even higher local P_1_ concentrations present. The two *T*_1_ time constants for the powder were about 100 μs and 1.3 ms.

**Table 1:** Fit Parameters for Hahn-Echo Low-Field Pulse-EPR Experiments^a^

	*M*_1_	*T*_2_^1^ (μs)	*M*_2_	*T*_2_^2^ (μs)	*A*
powder	8(1)	1.9(1)	0.30(9)	10(2)	0.006(4)
single crystal	3.2(9)	2.2(5)	0.2(1)	18(8)	0.01(1)

a Uncertainties indicate 95% confidence intervals. The fitting equations are given in section 2.4.3.

**Table 2:** Fit Parameters for Inversion Recovery Low-Field Pulse-EPR Experiments^a^

	*M*_1_	*T*_1_^1^ (ms)	*M*_2_	*T*_1_^2^ (ms)	α (rad)
powder	0.41(4)	0.11(2)	0.58(4)	1.3(1)	4.33(1)
single crystal	0.25(5)	0.07(3)	0.74(5)	1.3(2)	4.40(3)

a Uncertainties indicate 95% confidence intervals. The fitting equations are given in section 2.4.3.

This heterogeneity is key to understanding how the different mechanisms observed above appear to coexist in the same sample. In regions with low P1 concentration, the relatively isolated defects exhibit DNP via the solid effect. As the P1 concentration increases, we see the appearance of the CE in those crystallites where the orientation permits the cross effect condition to be satisfied. Some of the local P1 clusters become fast-relaxing sites that are then responsible for the appearance of the truncated cross effect. As these cluster resonances can be fairly broad,^74^ it is possible to satisfy the truncated cross effect conditions at most of the crystal orientations. If the P1 in the clusters are close enough for exchange interactions to become important, this could also explain the origin of the Overhauser effect. Overhauser DNP has previously been observed in graphite^66^ as well as in exchange-coupled donor clusters in silicon.^54^ However, a more systematic study of the DNP spectrum as a function of magnetic field and temperature is needed to uniquely identify the underlying mechanisms for the observed Overhauser effects, especially in light of the different signs observed for this effect in different experimental regimes.

## 4 Conclusions

In summary, we have shown substantial ^13^C-DNP enhancement (>100) with P1 centers in diamonds at room temperature. We have also shown that the DNP enhancement proceeds via a complex combination of SE, OE, CE, and tCE DNP mechanisms, all within the same sample. The DNP enhancements observed are limited by the available power and could be improved by going to higher power and potentially higher magnetic field. Additionally, it might be possible to hyperpolarize the P1 centers via their interactions with adjacent optically polarized NV centers which could dramatically improve DNP enhancements even further.^75,76^

Using DNP to increase the sensitivity of NMR has the potential to significantly expand the application of this versatile spectroscopic technique to ever-smaller samples. This technique will also be useful for enhancement of NMR signals in NV-detected NMR at a high field^77^ and be potentially applicable to NV-detected NMR of external spins. Hyperpolarized NMR of nano- and microdiamond tracers could prove valuable both in biomedical applications and in fluid engineering. It should also be possible to transfer the hyperpolarization to spins external to the diamond via spin diffusion^78^ or cross-polarization.^29^ DNP with diamond chips would enable the use of magnetic resonance to study oriented low-dimensional systems such as thin films and 2D materials like graphene and its functional derivatives, transition-metal dichalcogenides, and 2D conductive metal–organic frameworks. These materials are increasingly being used as catalysts and chemiresistive devices and may hold promise for the development of novel quantum materials.

## Supporting Information Available

Millimieter wave system, characterization of ^13^C decoherence time, DNP enhancement of the single crystal, simulating the EPR spectrum, fitting the experimental DNP spectrum, buildup of the thermal ^13^C signal, power dependence of DNP, DNP buildup times, simulation of DNP mechanisms, and low-field pulse-EPR spectrometer and data processing (PDF)

## Notes

The data can be provided by the authors pending scientific review and a completed material transfer agreement. Requests for the data should be submitted to Daphna Shimon or Chandrasekhar Ramanathan.
