# Supplementary material for: Large Room Temperature Bulk DNP of 13C via P1 Centers in Diamond
Source: J Phys Chem C Nanomater Interfaces. 2022 Oct 3;126(41):17777–87. doi: 10.1021/acs.jpcc.2c06145 (PMC9589901; doi:10.1021/acs.jpcc.2c06145)
Supplement: Supplementary file 1 — jp2c06145_si_001.pdf [file jp2c06145_si_001.pdf]

Supporting Information for Publication: Large Room Temperature Bulk DNP of ^{13}C via P1 Centers in Diamond

Daphna Shimon,[1,*] Kelly A. Cantwell,[2] Linta Joseph,[2] Ethan Q. Williams,[2]
Zaili Peng,[3] Susumu Takahashi,[3,4] and Chandrasekhar Ramanathan[2,†]

[1]*Institute of Chemistry, The Hebrew University of Jerusalem, Edmond J. Safra, Givat Ram, Jerusalem 9190401, Israel.*
[2]*Department of Physics and Astronomy, Dartmouth College, Hanover, NH 03755, U.S.A.*
[3]*Department of Chemistry, University of Southern California, Los Angeles, CA 90089, U.S.A.*
[4]*Department of Physics and Astronomy, University of Southern California, Los Angeles, CA 90089, USA*

I. MILLIMETER WAVE SYSTEM

An Agilent 33220A arbitrary waveform generator (AWG) was used to create the waveforms used for continuous wave (CW) MW irradiation. The AWG output was set to 2.5 V, which was input into a Mini Circuits voltage controlled oscillator ZX95-1480+ (VCO) for CW or chirped excitation around a central frequency of 1.012 GHz. The 1.012 GHz output of the VCO was first mixed (Marki T3 mixer) with a variable frequency signal from the ($\sim$ 4 GHz) Quonset Microwave QM2010-4400 source to yield sidebands at $\sim$ 5 GHz and $\sim$ 3 GHz. The 3 GHz component mixer was filtered out using a Mini Circuits high-pass filter VHF-3500+ that only allows frequencies above 3.9 GHz to pass through. The variable frequency signal around 5 GHz was then mixed with an 88.56 GHz phase-locked oscillator source to reach 94 GHz, and filtered again to remove the low frequency sideband, using an iris filter as shown in Figure S1. This is a modification of the Millitech MW bridge previously described in [1]. In order to increase the available millimeter wave power we removed the voltage controlled attenuator (1.5 dB insertion loss) and the directional coupler (1.8 dB insertion loss) to get a maximum output power of 23.8 dBm (240 mW).

In order to vary the power below 240 mW we connected a variable 30 dB attenuator to the output to the source. In order to run the experiment at higher power, we modified a commercial 500 mW injection-locked 94 GHz source from Quinstar. We removed the varactor-tuned Gunn diode orignally used for the injection locking from the source and instead used the attenuated output of the Millitech source. We were unable to verify if the efficiency of injection-locking was uniform across the entire sweep bandwidth.

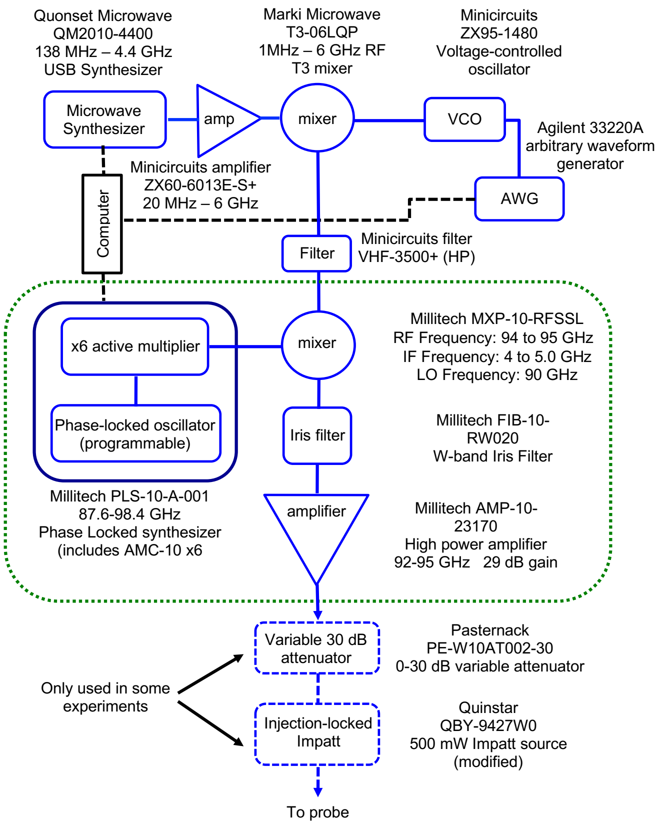

Figure S1. Schematic of the millimater wave source used in these experiments. Adapted with permission from Guy, M. L.; Zhu, L.; Ramanathan, C. *J. Magn. Res.* **2015**, 261, 11–18. Copyright 2015 ELSEVIER.

II. ^{13}C DECOHERENCE TIME

The NMR peak has a width of 1.12 kHz. This resonance shows significant inhomogeneous line-broadening and it is possible to detect several hundred echoes in a pulsed spin-lock experiment (using a train of $\pi/2$ pulses [2]) as shown in Figure S2. Stroboscopic detection of multiple echoes would allow a significant *additional* improvement in the signal-to-noise ration (SNR) obtained. The decay times measured are significantly shorter than those recently measured by Beatrez *et al.* [2], potentially due to the microwaves being continually on during the experiment.

III. DNP ENHANCEMENT OF THE SINGLE CRYSTAL

We tried to measure the thermal equilibrium ^{13}C signal from the single crystal diamond sample. Figure S3 shows average of 800 scans of the thermal signal measured with a relaxation

* daphna.shimon@mail.huji.ac.il
† chandrasekhar.ramanathan@dartmouth.edu

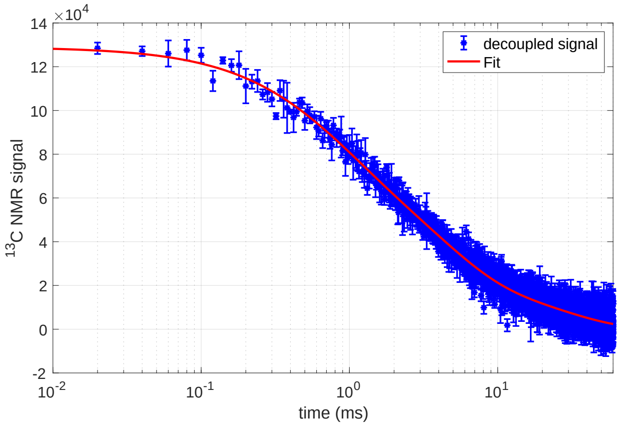

Figure S2. The decay of a pulsed spin-locking echo train with a 10 μs τ spacing following hyperpolarization for 60 s. The data were collected in a two-dimensional experiment. The best fit is obtained with a 3-component fit with time constants of 25.5 ± 1.5 ms, 3.8 ± 0.4 ms and 0.69 ± 0.13 ms with relative intensities of 0.19, 0.47 and 0.33.

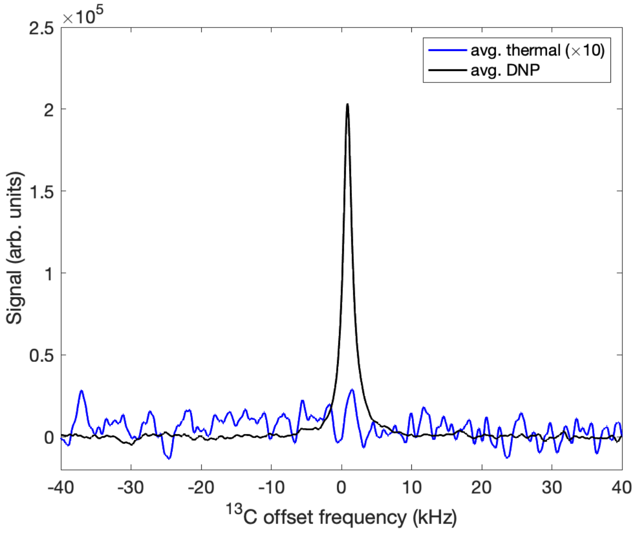

Figure S3. Thermal and DNP-enhanced ^{13}C NMR spectra from the single-crystal diamond sample. The thermal signal is an average of 800 scans and has been vertically scaled by a factor of 10 to better visualize the signal. The DNP signal is an average of 8 scans and is acquired with the crystal at the orientation shown in Figure S4.

delay of 500 s as well as the average of 8 scans of the DNP signal measured at 93.77 GHz with the same buildup of 500 s with about 240 mW of microwave power. Since we are unable to see the thermal ^{13}C signal, we use the SNR of the DNP signal to estimate a minimum DNP enhancement of 180 for the single crystal sample. Note the thermal signal was acquired with a receiver gain that was 4 times larger than that used in the DNP experiment.

In Figure S4 we show a fit of a single crystal at a second orientation, using the method described in the main text, and below.

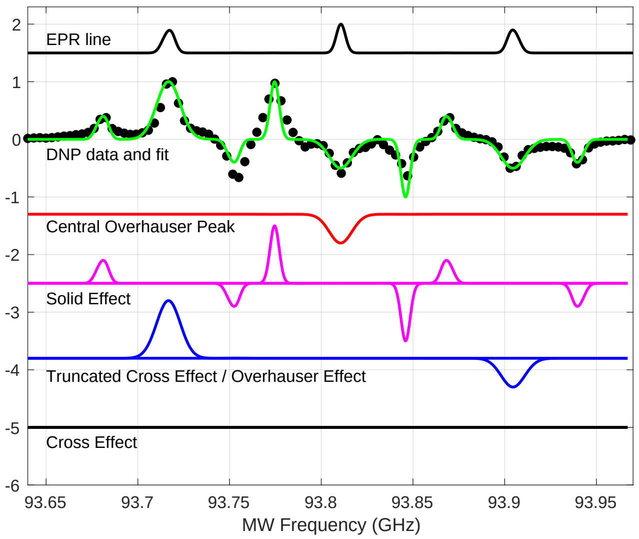

Figure S4. Fit of experimental constant-frequency DNP spectrum (black circles) for a single-crystal diamond sample. The fit uses a sum (green line) of the OE (red line), SE (magenta line), the truncated CE (blue line), and the CE (black line). The EPR line plotted above includes line broadening of 10 MHz of the outer EPR lines. The single crystal EPR line was simulated with EasySpin [3] using the Euler angles $\alpha = 136.8000°$, $\beta = 129.7918°$, $\gamma = 309.6000°$.

IV. SIMULATING THE EPR SPECTRUM

Given the concentration of P1 centers it is also important to account for magnetic dipolar interactions between electron spins in the crystal. Without knowing the specific distribution of P1 centers in the system we assume here that the P1 centers are uniformly distributed. We used a Monte Carlo simulation to randomly seed a diamond lattice with a 100 ppm concentration of P1 centers and estimated the pairwise distribution of dipolar couplings in the sample by averaging over a large number of configurations.

We used a lattice with $15 \times 15 \times 15$ unit cells, each with 2 atoms per unit cell and randomly seeded the sites with P1 centers using a binomial probability determined by the concentration. The distribution of pairwise P1 dipolar interactions was then calculated and stored. We averaged this distribution over 10,000 random lattice configurations to estimate the distribution of pairwise dipolar couplings in the system as shown in Figure S5.

We then used EasySpin package [3] to perform quantum mechanical simulations of pairs of P1 centers . As noted earlier, each P1 center was modeled as an e-^{14}N system, with an isotropic g=2.0024, hyperfine coupling strengths with the ^{14}N nucleus of $A_x^N = A_y^N$ = 82 MHz, A_z^N = 114 MHz [4]. The ^{14}N nuclear quadrupolar interaction was neglected. EasySpin automatically computes the powder average of the spectrum and uses the 227 (F d -3 m) space group symmetry of the diamond lattice.

For the single crystal simulation shown in Figure 4 of the main text, we simulated a single crystal orientation with Eu-

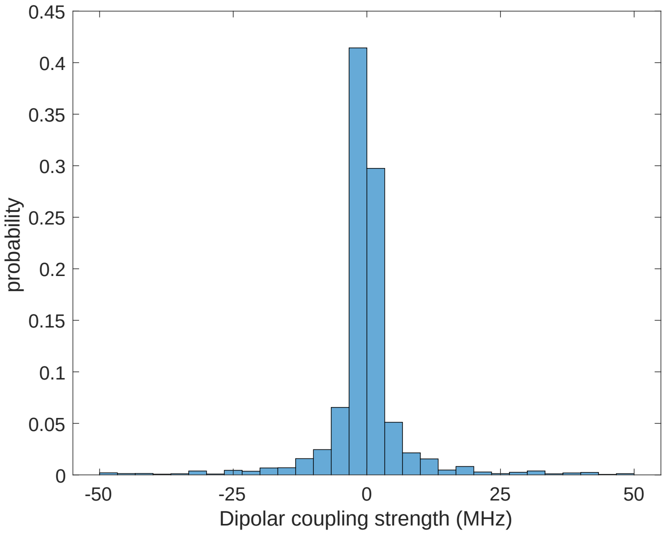

Figure S5. Simulated distribution of pairwise dipolar couplings for a 100 ppm P1 concentration diamond sample.

ler angles $\alpha = 54.0013°$, $\beta = 134.4274°$, $\gamma = 18.0023°$, and the same space group, isotropic g-value and hyperfine interactions described in the previous paragraph. For the single crystal simulation shown in Figure S4 we simulated a single crystal orientation with Euler angles $\alpha = 136.8000°$, $\beta = 129.7918°$, $\gamma = 309.6000°$.

V. FITTING THE EXPERIMENTAL DNP SPECTRUM

In the main text we fit the DNP spectrum using a crude method of convoluting the EPR line with delta functions to form the basic shapes for the solid effect (SE), Overhauser effect (OE) (same shape used for tCE) and cross effect (CE) DNP mechanisms, and changing their amplitude to achieve the best agreement with the experimental spectrum. This method was previously described in Banerjee et al. [5].

In order to do this, the EPR line, $g(\omega_{\text{MW}})$, was split into three ranges:

- The low frequency line: $g_1(\omega_{\text{MW}})$ for $93.56 \leq \omega_{\text{MW}} \leq 93.7740$ GHz
- The central line: $g_2(\omega_{\text{MW}})$ for $93.7740 < \omega_{\text{MW}} < 93.8590$ GHz
- The high frequency line: $g_3(\omega_{\text{MW}})$ for $93.8590 \leq \omega_{\text{MW}} \leq 94.0610$ GHz

The EPR line used for the powder DNP spectrum was simulated using using a e-^{14}N e-^{14}N spin system, averaged over 100 of the most probable e-e dipolar interactions between the two systems (see Figure S5). This averaging caused a slight broadening of the two external EPR lines (as explained above). For the single crystal DNP spectrum, we simulated a single crystallite orientation, again using EasySpin.

In order to mimic the effects of the effective MW irradiation strength on the off-resonance irradiation, for each DNP mechanisms, we added Gaussian broadening to the different OE/tCE, SE and CE lineshapes [6]. The effective irradiation on the SE is weakest, and therefore it was broadened with a Gaussian function with a FWHM of 0.1 MHz. The effective irradiation of the OE, CE and tCE are much larger, and therefore they were broadened with a Gaussian function with a FWHM of 8 MHz. The FWHM values were chosen in order to give the best fit for the DNP spectrum. However, from perturbation theory we know that the effective irradiation at the DQ/ZQ transition (as in the SE) is of the order of about 1/100 of the irradiation on a single quantum transition (as in the CE/OE/tCE), which is reflected in the values chosen [7, 8].

The basic DNP shapes used for the fitting were calculated by convoluting the three EPR lines with delta functions at the appropriate frequency separations. The OE shapes are just the EPR lines:

$$\text{OE}_1(\omega_{\text{MW}}) = g_1(\omega_{\text{MW}}) \tag{1}$$

$$\text{OE}_2(\omega_{\text{MW}}) = -g_2(\omega_{\text{MW}}) \tag{2}$$

$$\text{OE}_3(\omega_{\text{MW}}) = -g_3(\omega_{\text{MW}}) \tag{3}$$

The SE shapes are just the EPR lines convolved with delta functions at $\omega = \omega_{\text{MW}} + \omega_C$ and $\omega = \omega_{\text{MW}} - \omega_C$, giving:

$$\text{SE}_1(\omega_{\text{MW}}) = g_1(\omega_{\text{MW}} + \omega_C) - g_1(\omega_{\text{MW}} - \omega_C) \tag{4}$$

$$\text{SE}_2(\omega_{\text{MW}}) = g_2(\omega_{\text{MW}} + \omega_C) - g_2(\omega_{\text{MW}} - \omega_C) \tag{5}$$

$$\text{SE}_3(\omega_{\text{MW}}) = g_3(\omega_{\text{MW}} + \omega_C) - g_3(\omega_{\text{MW}} - \omega_C) \tag{6}$$

The CE shapes are just the EPR lines convolved with delta functions at $\omega = \omega_{\text{MW}} + \omega_C$ and $\omega = \omega_{\text{MW}} - \omega_C$, and then convolved again with the two outer EPR lines, giving:

$$\text{CE}_1(\omega_{\text{MW}}) = g_1(\omega_{\text{MW}}) * [g_1(\omega_{\text{MW}} + \omega_C) - g_1(\omega_{\text{MW}} - \omega_C)] \tag{7}$$

$$\text{CE}_3(\omega_{\text{MW}}) = g_3(\omega_{\text{MW}}) * [g_3(\omega_{\text{MW}} + \omega_C) - g_3(\omega_{\text{MW}} - \omega_C)] \tag{8}$$

CE from the central EPR line was not considered because the line is not broad enough to fulfill the CE-condition of two electrons separated by the nuclear Larmor frequency.

To reproduce the experimental DNP spectrum, a linear combination of the different basic DNP shapes was calculated:

$$\epsilon(\omega_{\text{MW}}) = \epsilon_{\text{OE}}(\omega_{\text{MW}}) + \epsilon_{\text{SE}}(\omega_{\text{MW}}) + \epsilon_{\text{CE}}(\omega_{\text{MW}}) \tag{9}$$

where

$$\begin{aligned}\epsilon_{\text{OE/tCE}}(\omega_{\text{MW}}) &= \epsilon_{\text{OE},1}(\omega_{\text{MW}}) + \epsilon_{\text{OE},2}(\omega_{\text{MW}}) + \epsilon_{\text{OE},3}(\omega_{\text{MW}}) \\ &= k_{\text{OE},1}\text{OE}_1(\omega_{\text{MW}}) + k_{\text{OE},2}\text{OE}_2(\omega_{\text{MW}}) + \\ &\quad k_{\text{OE},3}\text{OE}_3(\omega_{\text{MW}})\end{aligned} \tag{10}$$

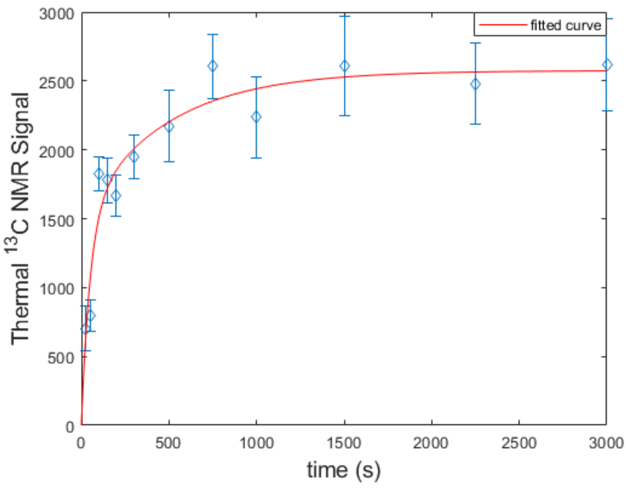

Figure S6. Thermal build up of NMR signal (blue diamonds) overlaid with the biexponential fit (red line).

$$\epsilon_{\text{SE}}(\omega_{\text{MW}}) = \epsilon_{\text{SE},1}(\omega_{\text{MW}}) + \epsilon_{\text{SE},2}(\omega_{\text{MW}}) + \epsilon_{\text{SE},3}(\omega_{\text{MW}})$$
$$= k_{\text{SE},1}\text{SE}_1(\omega_{\text{MW}}) + k_{\text{SE},2}\text{SE}_2(\omega_{\text{MW}}) + k_{\text{SE},3}\text{SE}_3(\omega_{\text{MW}}) \quad (11)$$

$$\epsilon_{\text{CE}}(\omega_{\text{MW}}) = \epsilon_{\text{CE},1}(\omega_{\text{MW}}) + \epsilon_{\text{CE},3}(\omega_{\text{MW}})$$
$$= k_{\text{CE},1}\text{CE}_1(\omega_{\text{MW}}) + k_{\text{CE},3}\text{CE}_3(\omega_{\text{MW}}) \quad (12)$$

In order to fit the DNP spectrum we manually adjusted the values of $k_{\text{OE},1}$, $k_{\text{OE},2}$, $k_{\text{OE},2}$, $k_{\text{SE},1}$, $k_{\text{SE},2}$, $k_{\text{SE},2}$, $k_{\text{CE},1}$ and $k_{\text{CE},2}$, and determined the best fit by eye. The contribution of the tCE to the fit is included in the OE terms for the two outer EPR lines, since the shapes are identical for both mechanisms.

VI. BUILDUP OF THE THERMAL ^{13}C SIGNAL

The buildup for the thermal signal (T_1 curve) was recorded using a saturation-recovery experiment. Because of the long times involved in this experiment, the longer times were recorded with fewer scans (64 instead of 128 or 480 scans), and therefore the intensity of these points have slightly larger error-bars. The curve was fitted with a biexponential fit, with time constants of $T_1^{\text{short}} = 51 \pm 29$ s and $T_1^{\text{long}} = 481 \pm 425$ s. Note that the uncertainties in the fit are quite large due to both the low SNR in the thermal experiment and the biexponential nature of the fit.

VII. POWER DEPENDENCE OF DNP

It has been reported that different DNP mechanisms have different dependence on the power of the MW irradiation. As such, we should be able to tell apart the SE, OE and CE via their power dependencies [9, 10]. Figure S7 shows the DNP spectrum as a function of applied MW power at buildup times of 300 s. For the 240 mW source, the power was varied by changing the settings of a variable attenuator. The figure suggests that the DNP enhancement grows linearly with power at all MW irradiation frequencies up to about 240 mW suggesting that we are in a low enough power regime that the enhancement has not reached saturation for any of the DNP mechanisms [11]. At 500 mW the SE contributions have continued to increase linearly but the enhancement starts to saturate at other MW frequencies.

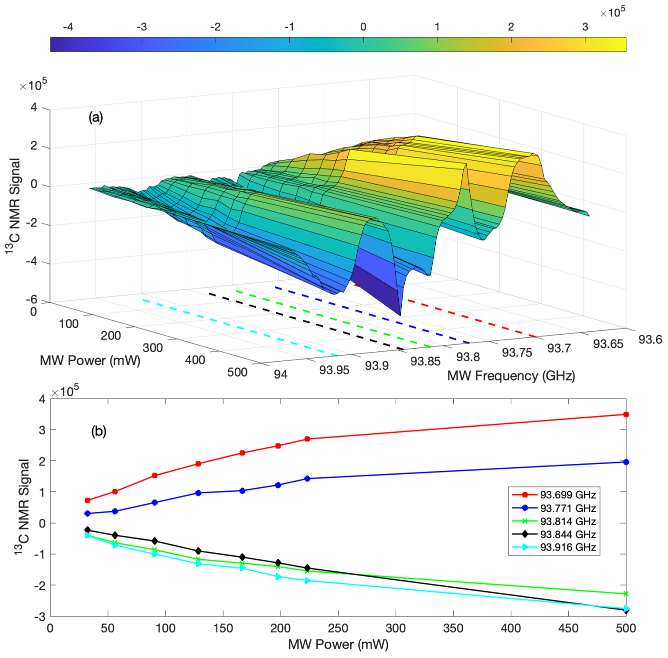

Figure S7. (a) ^{13}C NMR Signal as a function of MW power at a DNP buildup time of 300 s indicating that the DNP enhancement is still growing as a function of power at all frequencies in our experiment. (b) Power dependence at specific MW frequencies showing that the enhancement is beginning to saturate at some frequencies.

VIII. DNP BUILDUP TIMES

In addition, to compare the various DNP mechanisms, we also characterized the buildup time of the hyperpolarization as a function of MW frequency at 240 mW up to 3000 s as shown in Figure S8. Almost all the buildup curves could be fit with a two-component fit, T_{bu}^{short} and T_{bu}^{long}. This biexponential behavior suggests that the two components consist of ^{13}C spins directly hyperfine-coupled to an adjacent P1 center (short time constant) and more distant spins that rely on nuclear spin diffusion to mediate both T_1 relaxation and DNP buildup (long time constant) [12]. Only a single exponential fit was required at a few MW excitation frequencies. The fits fail when the DNP enhancement is low such as on the edges of the DNP spectrum and in locations where the enhancement

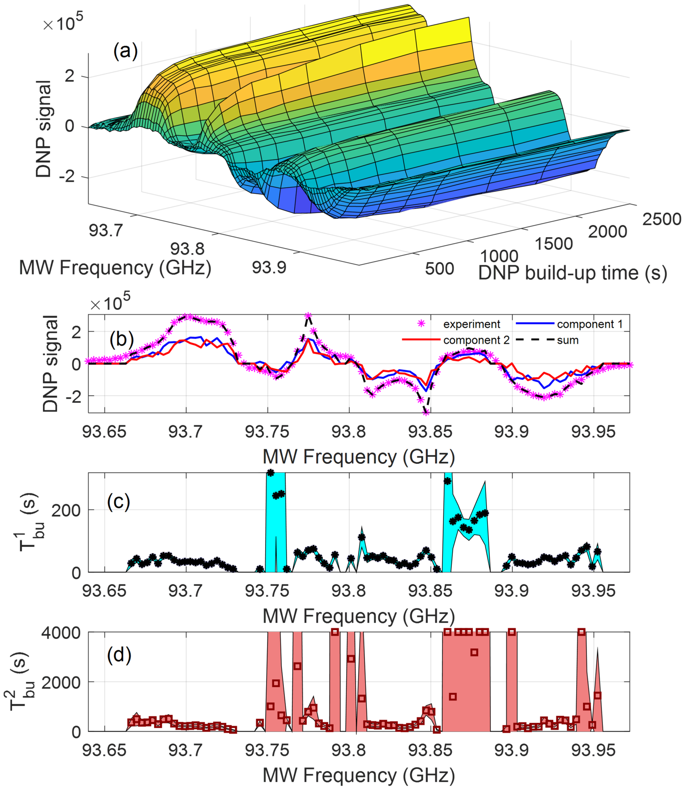

Figure S8. (a) Surface Plot showing the build up of the DNP spectrum as a function of MW irradiation time. (b) The buildup curves at each MW frequency were fit using a two-component fit. The amplitudes of the two components and their sum are shown in (b), while (c) and (d) show the best-fit short and long time constants as a a function MW frequency. The shaded region indicates the $\pm\sigma$ confidence interval of the fits. The fits fail when the DNP enhancement is low and where the enhancement switches sign. There is significant uncertainty associated with some of the longer time constants since data was only acquired up to a maximum buildup time of 3000 s.

switches sign. The amplitudes and time constants of the two components are also shown in the figure. The shaded region indicates the $\pm\sigma$ confidence interval of the fitted time constants. There is significant uncertainty associated with some of the longer time constant since data was only acquired up to a maximum buildup time of 3000 s.

At most MW excitation frequencies, the short component is on the order of 50 s and the long component on the order of 500 s – similar to the thermal buildup times. At most MW frequencies, the amplitudes of the short and long time constant components are nearly the same, except at the outer lobes around 93.7 GHz and 93.92 GHz, where the short time component is seen to be significantly larger than the long-time component. The buildup was observed to be particularly slow for the SE peaks.

The fact that $\mathrm{T}_1^{\mathrm{short}} \approx \mathrm{T}_{bu}^{\mathrm{short}}$ and $\mathrm{T}_1^{\mathrm{long}} \approx \mathrm{T}_{bu}^{\mathrm{long}}$ is another indication that we do not have enough MW power to drive the DNP at a rate that is faster than T_1 [7, 9].

IX. SIMULATION OF DNP MECHANISMS

We performed numerical simulations of the spin dynamics of two small spin systems, an e-^{14}N system and an e-^{14}N-^{13}C system. The simulations were based on the method described by the Vega group [6, 8, 13, 14]. Numerical simulations involving e-^{14}N and e-^{14}N-^{13}C systems are described in Shimon et al. in the context of the role the ^{14}N nucleus plays in DNP enhancement of spin half nuclei using Nitroxide radicals [6].

A. Simulation of the e-^{14}N System

We begin by looking at an e-^{14}N system, containing one electron that is strongly hyperfine coupled to a ^{14}N nucleus. In the MW rotating frame, the Hamiltonian of the system without the MW irradiation is given by:

$$H_0^N = \Delta\omega_e S_z - \omega_N I_z^N + A_z^N S_z I_z^N + A_x^N S_z I_x^N + A_y^N S_z I_y^N$$

where S and I^N are the electron and nitrogen spins, respectively. $\omega_e = \omega_e - \omega_{\mathrm{MW}}$ is the electron off-resonance (which is determined by the g-tensor), ω_N is the ^{14}N Larmor frequency. A_z^N is the secular part of the e-N hyperfine interaction (which includes both isotropic and anisotropic shifts) and A_x^N and A_y^N are the pseudo-secular terms of the dipolar hyperfine interaction (which is anisotropic). Here, we simulated each P1 center as an e-^{14}N system, with an isotropic g=2.0024 and hyperfine coupling strengths with the ^{14}N nucleus with principal axis components $A_{xx}^N = A_{yy}^N$ = 82 MHz, A_{zz}^N = 114 MHz [4]. A single crystal orientation was chosen, such that A_z^N = 98 MHz, and A_x^N = -16 MHz and A_y^N = 0 MHz. In the rotating frame, the MW irradiation term is

$$\omega_{\mathrm{MW}} = \omega_1 S_z. \tag{13}$$

Note, that the nuclear quadrupolar interaction is not included in these simulations. First, we diagonalize H_0^N according to

$$H_0^{Nd} = V^{-1} H_0^N V \tag{14}$$

The A_z^N term splits the EPR transition $|\uparrow_e\rangle \leftrightarrow |\downarrow_e\rangle$ into three transitions, according the nitrogen spin eigenstates $|\chi_N\rangle = |-1\rangle$, $|0\rangle$ or $|1\rangle$, such that the electron transitions can be written as $|\uparrow_e, \chi_N\rangle \leftrightarrow |\downarrow_e, \chi_N\rangle$, separated by A_z^N. The A_x^N and A_y^N terms result in weak state mixing of adjacent (i.e., $\Delta m_I = \pm 1$) nitrogen spin states within each electron manifold, such that:

$$|\chi_e, \pm\tilde{1}\rangle = c_N|\chi_e, \pm 1\rangle + s_N|\chi_e, 0\rangle \tag{15}$$

$$|\chi_e, \tilde{0}\rangle = c_N|\chi_e, 0\rangle + s_N|\chi_e, +1\rangle - s_N|\chi_e, -1\rangle \tag{16}$$

where $|\chi_e\rangle = |\uparrow_e\rangle$ or $|\downarrow_e\rangle$, $c_N = \cos\zeta_N$ and $s_N = \sin\zeta_N$, $\tan 2\zeta_N = \sqrt{(A_x^N)^2 + (A_y^N)^2}/[8(\omega_N + A_z^N)]$, if $\sqrt{(A_x^N)^2 + (A_y^N)^2} \ll \omega_N$ and A_z^N, from perturbation theory [15].

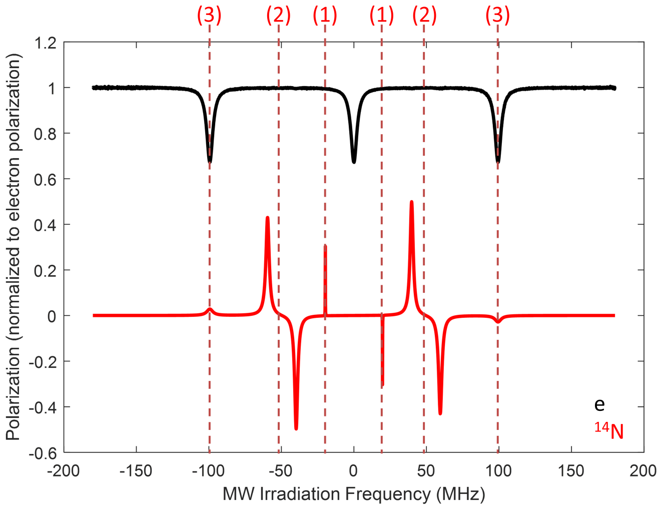

Figure S9. Simulated electron (black) and ^{14}N (red) polarizations as a function of the MW irradiation frequency for an e-^{14}N spin system. The polarizations are normalized according to the steady state electron polarization. The MW irradiation frequency is plotted in MHz for convenience, and is referenced to the electron Larmor frequency, ω_e =94 GHz. The simulation parameters: ω_e =94 GHz, ω_N =10 MHz, A_z^N =98 MHz, A_z^N =98 MHz, A_x^N=-16 MHz, A_y^N =0 MHz, ω_1 =0.5 MHz, T_{1e} =10^3 ms, T_{1N} =1 s, T_{2e} =10 μs and T_{2N} =100 μs. No cross relaxation was added to the system.

Next, we transform the MW irradiation term to the same frame, according to

$$H^d_{\text{MW}} = V^{-1} H_{\text{MW}} V \quad (17)$$

The weak state mixing described above also results in weak effective irradiation between double quantum and zero quantum transitions, which give the ^{14}N-SE-DNP enhancement. The strength of the effective irradiation is proportional to the state-mixing, such that it is $\omega^N_{1,\text{eff}} = |s_N|\omega_1$ for the ^{14}N-SE-DNP and even weaker for second order ^{14}N-SE-DNP effects.

In Figure S9 we plot the steady state ^{14}N nuclear polarization, $P_N(\omega_{\text{MW}})$, and the electron polarization, $P_e(\omega_{\text{MW}})$, during MW irradiation as a function of the frequency of the MW irradiation. Here we normalize all electron and nuclear polarizations to the value of the electron polarization at thermal equilibrium, $P_e^{\text{eq}} = 1$ (i.e., when no MW irradiation is applied). Concentrating on the electron, it is clear that when the MW irradiation frequency is far off resonance the electron polarization is at its largest value. When the MW irradiation is on-resonance with one of the electron transitions the electron polarization is reduced (i.e., the electron is partially saturated). Far off resonance $P_N(\omega_{\text{MW}}) = P_N^{\text{eq}} \approx 0$ because the nuclear polarization is negligible compared to the electron polarization ($P_e^{\text{eq}} \gg P_N^{\text{eq}}$). At certain frequencies the $P_N(\omega_{\text{MW}}) \neq 0$ is enhanced due to the small state mixing between the nitrogen spin states, which is a result of the A_z^N term of the hyperfine interaction.

Concentrating on the ^{14}N-DNP spectrum in Figure S9, it can be clearly seen that there are three distinct DNP features (marked 1,2 and 3 in the figure):

1. Two narrow features appearing at $\omega \approx \omega_e \pm 2\omega_N$ are second order SE-DNP transitions. Positive enhancement is achieved when irradiating on the $|\alpha_e, \tilde{1}\rangle \leftrightarrow |\beta_e, -\tilde{1}\rangle$ transition, and negative enhancement is achieved when irradiating between $|\alpha_e, -\tilde{1}\rangle \leftrightarrow |\beta_e, \tilde{1}\rangle$.

2. Four broader features appearing at $\omega = \omega_e \pm \omega_N \pm A_z^N/2$ and $\omega = \omega_e \pm \omega_N \mp A_z^N/2$ are the SE transitions of the central EPR line, where the separation between positive and negative enhancement is $2\omega_N + A_z^N$ or $2\omega_N - A_z^N$. The outer SE pair is formed as a result of irradiation on the $|\alpha_e, \tilde{0}\rangle \leftrightarrow |\beta_e, -\tilde{1}\rangle$ and $|\alpha_e, \tilde{0}\rangle \leftrightarrow |\beta_e, +\tilde{1}\rangle$ transitions. The inner SE pair is formed as a result of irradiation on the $|\alpha_e, -\tilde{1}\rangle \leftrightarrow |\beta_e, \tilde{0}\rangle$ and the $|\alpha_e, \tilde{1}\rangle \leftrightarrow |\beta_e, \tilde{0}\rangle$. The two SE pairs are centered around ω_e, but they have opposite signs, such that signs of the enhancement of the inner SE pair are opposite of those of the outer SE pair.

3. The two broader features at $\omega = \omega_e \pm A_z^N$ appear on resonance on the electron, as one would expect from the Overhauser effect, but are in fact due to the SE. In this case, the state-mixing described in Equations 15-16 results in electron-nitrogen cross-relaxation terms that connect the nitrogen states within each electron manifold. When irradiating on the low frequency electron line (i.e. on the $|\alpha_e, -\tilde{1}\rangle \leftrightarrow |\beta_e, -\tilde{1}\rangle$ transition) and then positive enhancement is achieved. In a similar manner, when irradiating on the high frequency electron line (i.e. on the $|\alpha_e, +\tilde{1}\rangle \leftrightarrow |\beta_e, +\tilde{1}\rangle$ transition), T_{1ZQ} forms on the $|\alpha_e, +\tilde{1}\rangle \leftrightarrow |\beta_e, \tilde{0}\rangle$ transition and then negative enhancement is achieved. At the center of the DNP spectrum (when irradiating directly at ω, no enhancement is achieved).

B. Simulation of the e-^{14}N-^{13}C system

Next, we add a ^{13}C spin to the system described above. The Hamiltonian of this system is given by:

$$H_0^{NC} = H_0^N - \omega_C I_z^C + A_x^C S_z I_x^C + A_y^C S_z I_y^C$$

where I^C is the carbon spin operator, ω_C is the ^{13}C Larmor frequency and A_x^C and A_y^C are the pseudo-secular terms of the dipolar hyperfine interaction. The other terms were defined above. Note that no e-C secular hyperfine or N-C dipolar interactions were added to the simulation. Here, $A_x^C = A_y^C$.

Again we diagonalize the Hamiltonian H_0^{NC}, and then transform H_{MW} to the same frame, as described above. The $A_x^C = A_y^C$ term results in weak state mixing of carbon spin spin states within each electron-nitrogen manifold, such that:

$$|\chi_e, \tilde{\chi}_N, \alpha_C^*\rangle = c_C|\chi_e, \tilde{\chi}_N, \alpha_C\rangle + s_C|\chi_e, \tilde{\chi}_N, \beta_C\rangle \quad (18)$$
$$|\chi_e, \tilde{\chi}_N, \beta_C^*\rangle = c_C|\chi_e, \tilde{\chi}_N, \beta_C\rangle - s_C|\chi_e, \tilde{\chi}_N, \alpha_C\rangle \quad (19)$$

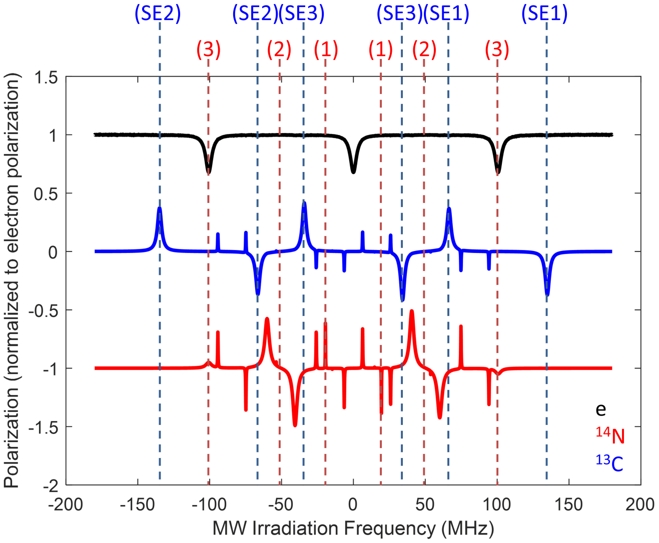

Figure S10. Simulated electron (black), ^{14}N (red) and ^{13}C polarizations as a function of the MW irradiation frequency for an e-^{14}N-^{13}C spin system. The polarizations are normalized according to the steady state electron polarization. The ^{14}N DNP spectrum was shifted in the y-axis for clarity. The MW irradiation frequency plotted in MHz for convenience, and is referenced to the electron Larmor frequency, ω_e =94 GHz. The simulation parameters: $\Delta\omega_e$ =94 GHz, ω_N =10 MHz, ω_C =34 MHz, A_z^N =98 MHz, A_x^N=-16 MHz, A_y^N =0 MHz, A_x^C = A_y^C =5.4 MHz (corresponds to a 15.4 nm distance), ω_1 =0.5 MHz, T$_{1e}$ =10^3 ms, T$_{1N}$ =1 s, T$_{1C}$ =100 s, T$_{2e}$ =10 μs and T$_{2N}$ =T$_{2C}$ =100 μs. No cross relaxation was added to the system.

where $\chi_e = |\alpha_e\rangle$ or $|\beta_e\rangle$, $\tilde{\chi}_N = |\pm\tilde{1}\rangle$ or $\tilde{\chi}_N = |\tilde{0}\rangle$ and $c_C = \cos\zeta_C$ and $s_C = \sin\zeta_C$ and $\tan 2\zeta_N = \sqrt{(A_x^C)^2 + (A_x^C)^2}/[8\omega_C]$, if $\sqrt{(A_x^C)^2 + (A_x^C)^2} \ll \omega_N$, from perturbation theory [15].

The weak carbon state-mixing described above also results in weak effective-irradiation on the e-C DQ and ZQ transitions, which results in ^{13}C-SE-DNP enhancement, as can be seen in Figure S10. The strength of the effective irradiation on the ^{13}C-SE transitions is proportional to the state-mixing, such that it is $\omega_{1,\text{eff}}^C = |s_C|\omega_1$ for the ^{13}C-SE-DNP and even weaker for second order ^{13}C-SE-DNP effects.

As can be seen in Figure S10, the addition of the ^{13}C nucleus only slightly alters the ^{14}N-DNP spectrum. The same three features described above are still visible here (marked 1,2 and 3 in the figure), in addition to several new and narrow DNP features. The additional narrow features are a result of second-order SE DNP lines involving the electron, the ^{14}N and the ^{13}C nuclei. For example, MW irradiation on the transition $|\alpha_e, \tilde{1}, \alpha_C^*\rangle \leftrightarrow |\beta_e, \tilde{0}, \beta_C^*\rangle$ will result in DNP enhancement of both the nitrogen and the carbon. As such, both the ^{14}N and the ^{13}C nuclei show DNP enhancement at these SE mechanisms. These effects are beyond the scope of the current work.

In addition to the narrow second-order SE DNP lines, the ^{13}C-DNP spectrum exhibits three ^{13}C-SE pairs, one from each electron line (marked SE1, SE2 and SE3 in the figure). These SEs appear at $\omega = \Delta\omega_e + A_z^N \pm \omega_C$ (SE1), $\omega = \Delta\omega_e - A_z^N \pm \omega_C$ (SE2) and $\omega = \Delta\omega_e \pm \omega_C$ (SE3). The ^{13}C-SE-DNP mechanisms result in positive enhancement when irradiating on the $|\beta_e, \tilde{\chi}_N, \alpha_C^*\rangle \leftrightarrow |\alpha_e, \tilde{\chi}_N, \beta_C^*\rangle$ transition and negative enhancement when irradiating on the $|\beta_e, \tilde{\chi}_N, \beta_C^*\rangle \leftrightarrow |\alpha_e, \tilde{\chi}_N, \alpha_C^*\rangle$ transition, for $\tilde{\chi}_N = -\tilde{1}$, $\tilde{0}$ or $\tilde{1}$.

In summary, the DNP simulations for an e-^{14}N-^{13}C system exhibit a ^{13}C-SE for each of the three e-^{14}N manifolds. In each case, the ^{13}C-SE is independent of the ^{14}N spin state, and does not involve the ^{14}N spin. Using this observation, we justify the convolution method of simulating the ^{13}C-DNP mechanisms (OE, SE and CE) for each e-^{14}N manifold separately (described above). Higher order ^{13}C-DNP enhancement features that involve both the ^{14}N spin as well as the ^{13}C spin are much more sensitive to the MW irradiation strength, and therefore, we are able to neglect them in our analysis of the experimental results.

X. LOW-FIELD PULSE-EPR SPECTROMETER AND DATA PROCESSING

The resonance frequency of 2.5 GHz is achieved by mixing a 2.476 GHz local oscillator with the output from a Tektronix AWG 7052 which is used to define pulse waveforms on a 24 MHz carrier. The pulses are amplified to approximately +27 dBm, then transmitted through a circulator to an antenna. At the end of the coax line is an exposed wire which capacitively couples to a shielded loop-gap resonator [16] (inspired by a previously published design [17]) that is 1.27 cm long and has an inner diameter of 0.73 cm. The received echo signal is amplified, mixed with the local oscillator and sampled at 400 MHz with a digitizer. In-phase and quadrature components of the pulses and echoes are transmitted and received, respectively, via IQ mixers. On-board averaging is performed on the digitizer, which returns to the lab computer the averaged echo waveform for a given set of experiment parameters. For each set of experiment parameters, the phase of the pulses is cycled through 0, $\pi/2$, π, and $3\pi/2$, and the resulting waveforms are phase shifted and added constructively in post-processing, thereby averaging out phase-independent systematic noise. The echo is then multiplied by a 1.0 μs Blackman window to suppress noise outside of the echo in the time-domain buffer. Next, it is demodulated to baseband and Fourier transformed. The amplitude is obtained by integrating over the Fourier transform of the echo with an integration range of 4.0 MHz. The in-phase and quadrature components of the integrated signal are the real and imaginary components of the echo amplitude. The uncertainties are obtained by calculating the standard deviation of the Fourier transformed buffer in the 2 MHz ranges above and below the 4 MHz range used to obtain the integrated signal, then multiplying by the square root of the number of points in the signal integration part of the buffer.

In the Hahn-echo experiments, the values of τ were chosen such that values of $\log_{10}(\tau)$ had a uniform random distribution, and the order in which they were performed was random as well. For the diamond powder, τ ranged from 1.95 μs to

98.1 μs. The lengths of the $\pi/2$ and π pulses were 200 ns and 400 ns respectively. For the single crystal macle-cut diamond, two data sets were combined: one with 28 points with τ ranging from 1.445 μs to 99.8065 μs and one with 37 points with τ ranging from 1.445 μs to 999.63 μs. The lengths of the $\pi/2$ and π pulses were 250 ns and 500 ns respectively. The delay between the end of one trial and the beginning of the repeat of that trial was 10 ms. All echo magnitudes were normalized by dividing by the number of averages and then dividing by the maximum strength echo obtained with the powder. For each set of parameters, 2^{14} trials were averaged on-board the digitizer, and with the phase-cycling described above, the total number of averages is 2^{16}.

The inversion recovery experiments used similar randomization to that of the Hahn-echo experiments. The values of τ_1 were chosen such that values of $\log_{10}(\tau_1)$ had a uniform random distribution and random order. For each set of parameters tested, back-to-back tests are performed with the inverting pulse off (amplitude set to 0) and then on, giving echo amplitudes S_{off} and S_{on}, respectively. The same phase correction is applied to both by setting S_{off} to be purely real. The inversion recovery signal plotted in Figure 7(b) in the main text is $S_{\text{IR}} = \text{Re}[S_{\text{on}}]/\text{Re}[S_{\text{off}}]$. For the diamond powder and the single crystal, τ_1 ranged from 1 μs to 60 ms, and τ_2 was set such that the time between the middles of pulses 2 and 3 was 3 μs. The delay between the end of one trial and the beginning of the repeat of that trial was 100 ms. For the powder, the pulses were the same as described in the previous paragraph, but for the single crystal, a recalibration was performed which led to $\pi/2$ pulses of 330 ns and π pulses of 660 ns being used. The need for longer pulse duration for the single crystal is due to a different resonator and sample holder being used.

REFERENCES

(1) Guy, M. L.; Zhu, L.; Ramanathan, C. *J. Magn. Res.* **2015**, *261*, 11–18.

(2) Beatrez, W.; Janes, O.; Akkiraju, A.; Pillai, A.; Oddo, A.; Reshetikhin, P.; Druga, E.; McAllister, M.; Elo, M.; Gilbert, B., et al. *Phys. Rev. Lett.* **2021**, *127*, 170603.

(3) Stoll, S.; Schweiger, A. *J. Magn. Res.* **2006**, *178*, 42–55.

(4) Loubser, J.; van Wyk, J. *Rep. Prog. Phys.* **1978**, *41*, 1201.

(5) Banerjee, D.; Shimon, D.; Feintuch, A.; Vega, S.; Goldfarb, D. *J. Magn. Res.* **2013**, *230*, 212–219.

(6) Shimon, D.; Hovav, Y.; Feintuch, A.; Goldfarb, D.; Vega, S. *Phys. Chem. Chem. Phys* **2012**, *14*, 5729–5743.

(7) Hovav, Y.; Feintuch, A.; Vega, S. *J. Magn. Res.* **2010**, *207*, 176–189.

(8) Hovav, Y.; Feintuch, A.; Vega, S. *J. Magn. Res.* **2012**, *214*, 29–41.

(9) Kundu, K.; Mentink-Vigier, F.; Feintuch, A.; Vega, S. *eMagRes* **2019**, 295–338.

(10) Corzilius, B. *Annual review of physical chemistry* **2020**, *71*, 143–170.

(11) Siaw, T. A.; Leavesley, A.; Lund, A.; Kaminker, I.; Han, S. *J. Magn. Res.* **2016**, *264*, 131–153.

(12) Ramanathan, C. *Appl Magn Reson* **2008**, *34*, Publisher: Springer-Verlag, 409–421.

(13) Hovav, Y.; Feintuch, A.; Vega, S. *Chem. Phys.* **2011**, *134*, 074509.

(14) Hovav, Y.; Feintuch, A.; Vega, S.; Goldfarb, D. *J. Magn. Res.* **2014**, *238*, 94–105.

(15) Schweiger, A.; Jeschke, G., *Principles of pulse electron paramagnetic resonance*; Oxford University Press on Demand: 2001.

(16) Froncisz, W.; Hyde, J. S. *J. Magn. Res.* **1982**, *47*, 515–521.

(17) Joshi, G.; Kubasek, J.; Nikolov, I.; Sheehan, B.; Costa, T.; Allao Cassaro, R.; Friedman, J. R. *Rev. Sci. Instrum.* **2020**, *91*, 023104.
